# Art therapy with children and adolescents experiencing acute or severe mental health conditions: A systematic review

**DOI:** 10.1177/00048674251361731

**Published:** 2025-09-08

**Authors:** Sarah Versitano, Stephanie Tesson, Chae-Weon Lee, Sheridan Linnell, Iain Perkes

**Affiliations:** 1 School of Social Sciences, Western Sydney University, Sydney, NSW, Australia; 2 Department of Psychological Medicine, Sydney Children’s Hospitals Network, Sydney, NSW, Australia; 3 School of Medicine & Dentistry, Griffith University, Brisbane, QLD, Australia; 4 Faculty of Medicine and Health, University of New South Wales, Sydney, NSW, Australia

**Keywords:** Art therapy, child, adolescent, mental health, systematic review

## Abstract

**Objective:**

Art therapy offers a predominantly non-verbal form of creative self-expression for people experiencing mental health issues. This systematic review aims to investigate the effectiveness and acceptability of art therapy for children and adolescents experiencing acute or severe mental health conditions.

**Methods:**

Following PRISMA guidelines, five electronic databases were searched (Embase, MEDLINE, Web of Science Core Collection, PsychINFO, CINAHL) using the search terms (‘art therap*’ OR ‘art psychotherap*’) AND (‘child*’ OR ‘adolescen*’ OR ‘youth’ OR ‘young’ OR ‘teen*’). Study quality was assessed based on methodological rigour, and narrative synthesis of findings was undertaken.

**Results:**

Of 3529 identified articles, 90 (23 original research papers and 67 case studies) met criteria. Art therapy delivery method, dose and intervention duration varied across studies. Nonetheless, synthesis of the 23 original research studies indicated high acceptability. Randomised control trials demonstrated effectiveness in reducing the severity of symptoms of post-traumatic stress disorder, depression and suicidal ideation. Quasi-experimental, cohort and cross-sectional studies also showed reductions in anxiety symptoms and improvements in emotion regulation, self-awareness, distress tolerance, confidence, communication and self-expression across various mental health conditions.

**Conclusion:**

Art therapy is an effective and acceptable treatment for young people experiencing acute or severe mental health conditions, with a preponderance of evidence for post-traumatic stress disorder. Effectiveness across conditions, settings and art therapy intervention type suggests the capacity of art therapy to adapt to needs of young people. Enhanced access to art therapy for young people navigating acute distress will support the provision of engaging and effective mental health treatments.

## Introduction

Mental health conditions are a major public health challenge for children and adolescents (Benton et al., 2021). The prevalence of mental health conditions in young people (Sara et al., 2023) and their significant impact on both functioning (Wille et al., 2008) and quality of life (Weitkamp et al., 2013) underscore the imperative for effective and acceptable therapeutic interventions (Kieling et al., 2011). While many young people respond positively to talk-based psychotherapies, some may not have the inclination or capacity to engage in these interventions owing to symptom severity, situational mutism or past negative experiences with talk-based therapies (Coholic et al., 2020; Versitano et al., 2024b).

Art therapy is a creative psychotherapeutic approach utilising art-making with visual and/or tactile media. Conducted within a therapeutic relationship with a trained therapist, it can support improved cognitive, physical and emotional well-being (American Art Therapy Association [AATA], 2023; Australian, New Zealand and Asian Creative Arts Therapies Association, 2024; British Association of Art Therapists [BAAT], 2024). Art therapy offers a non-verbal form of expression, which can be particularly useful for people who have difficulty articulating their emotions or experiences verbally (Chong, 2015; Rubin, 2005). Furthermore, art therapy can provide a safe and structured environment in which young people can explore overwhelming emotions, process traumatic experiences and establish coping mechanisms (Braito et al., 2022).

Art therapy has been used with children and adolescents experiencing mental health challenges such as depression, anxiety, post-traumatic stress disorder (PTSD), among other mental health conditions (Braito et al., 2022). Art therapy is also increasingly integrated into mental health services (Bosgraaf et al., 2020; Braito et al., 2022; Scope et al., 2017), warranting rigorous assessment of its effectiveness and acceptability. Clear assessment and evaluation of the effectiveness of art therapy with young people can be translated into advocacy for implementation in mental health services (Cornish, 2013).

Case studies have long supported the use of art therapy in mental health settings (e.g. Atlas et al., 1992; Diamond-Raab and Orrell-Valente, 2002; Naumburg, 1945) and reviews which explore the spectrum of art therapy practices and approaches have also been undertaken (Potash et al., 2016; Van Lith, 2016). Effectiveness has been demonstrated in reviews of art therapy with adult populations, highlighting improved mental health outcomes, beneficial psychosocial effects, as well as reductions in trauma and depression symptoms (Maujean et al., 2014; Schouten et al., 2015; Shukla et al., 2022). The effectiveness of art therapy with children has also been reviewed (Braito et al., 2022; Cohen-Yatziv and Regev, 2019; Moula, 2020) and improvements in quality of life, anxiety, self-concept, problem-solving skills, attitudes towards school, and emotional and behavioural difficulties have been shown, with evidence suggesting it is particularly beneficial for children who have experienced trauma, or who have PTSD symptoms (Braito et al., 2022). However, reviews which focused on art therapy for children and adolescents did not examine acceptability, and excluded non-English publications (Braito et al., 2022; Cohen-Yatziv and Regev, 2019; Moula, 2020). Further to this, previous reviews have also placed limitations on outcome measures used, excluding case studies and qualitative studies (Cohen-Yatziv and Regev, 2019; Moula, 2020); however, our current review will include both qualitative and quantitative research with no restriction regarding outcome measures used.

Our systematic review will address these gaps by including non-English publications, placing fewer limitations on outcome measures and exploring both effectiveness and acceptability of art therapy interventions, with a focus on young people experiencing acute or severe mental health conditions. Discerning acceptability is critical when considering the use of therapeutic interventions in mental health settings (Sekhon et al., 2017). This is particularly salient with young people, as engagement and sustained participation are often challenging, yet essential for positive outcomes (Shin and Ahn, 2023). Increased understanding of art therapy acceptability and effectiveness will assist in delineating efficacious treatments for young people in acute or severe phases of mental health care, which is relevant to the current growing mental health crisis (Benton et al., 2021).

## Methods

The protocol for this systematic review was registered on PROSPERO (CRD42024468934) and follows the guidelines for Preferred Reporting Items for Systematic Reviews and Meta-analyses (PRISMA) (Page et al., 2021).

### Search strategy

Existing peer-reviewed research published from database inception up until November 2023 was searched on Embase, MEDLINE, Web of Science Core Collection, PsychINFO & CINAHL. Search terms were (‘art therap*’ OR ‘art psychotherap*’) AND (‘child*’ OR ‘adolescen*’ OR ‘youth’ OR ‘young’ OR ‘teen*’). A secondary search was conducted before final analysis to include additional articles published up until 17 September 2024.

### Inclusion and exclusion criteria

Peer-reviewed articles reporting original research on art therapy for individuals aged 0–24 years during an acute and/or severe mental health phase were included. For non-English language articles which met all other inclusion criteria, Google translate software was used to facilitate data extraction (Jackson et al., 2019). Individuals in an acute phase of care were defined as those who were (1) admitted in an inpatient mental health setting; (2) experiencing active psychotic, manic or suicidal symptoms and/or (3) experiencing severe, extreme or sudden onset symptoms of any other psychiatric disorders, e.g., depression or anxiety (American Psychological Association [APA], 2018). Unless stipulated, all Diagnostic and Statistical Manual of Mental Disorders 5 (DSM-5-TR) psychiatric conditions, plus disorders reported in earlier DSM versions or the International Classification of Diseases 11 (ICD-11), were considered for inclusion based on symptom severity and/or acuity, e.g., obsessive compulsive disorders, functional neurological disorders, eating disorders or mood disorders. Pervasive neurodevelopmental conditions such as intellectual disability and autism spectrum disorder (ASD) were included where there were intercurrent severe or acute mental health challenges.

If mental health diagnosis and illness severity were ambiguous regarding the above criteria, then social and environmental factors were also considered: for instance, if participants had a confirmed or probable history of complex trauma and/or if they were in a high-risk group such as out-of-home care or juvenile justice. Such clinically ambiguous cases were considered for inclusion in consultation with a child and adolescent psychiatrist (I.P.). When available, pre-test scores were examined to gauge symptom severity according to scoring and clinical cut-offs. Both qualitative and quantitative research were included, with no restriction regarding outcome measures. Studies where art therapy was conducted in combination with other therapeutic interventions were included (e.g. art therapy and mindfulness), given an art therapist or qualified health professional facilitated them. Articles were excluded when (1) young people were not experiencing an acute or severe mental health condition and/or (2) an arts-based intervention was not identified by authors as ‘art therapy’ or ‘art psychotherapy’.

### Study selection and data extraction

Data from the searches conducted were imported and de-duplicated in EndNote. Remaining citations were imported into Covidence, where removal of duplicates was undertaken again by the first author (S.V.). Two authors (S.V., S.T.) screened titles and abstracts to identify relevant studies. Two authors (S.V. and C.L.) then undertook full-text review of the remaining studies. Discrepancies were resolved by consensus. Consultation with a co-author child and adolescent psychiatrist was undertaken as required to discern eligibility, particularly when symptom acuity was unclear (I.P.). Three authors extracted data from the included studies in Excel (S.V., C.L. and S.T.). The first author (S.V.) summarised information from the original research studies relevant to study aims, effectiveness and acceptability, and a co-author (S.T.) summarised case study findings separately.

### Quality assessment

Hawker et al.’s (2002) critical appraisal tool was used to assess research quality, scoring methodological rigour based on nine categories: (1) abstract and title, (2) introduction and aims, (3) method and data, (4) sampling, (5) data analysis, (6) ethics and bias, (7) findings/results, (8) transferability/generalisability, (9) implications and usefulness. Categories were independently scored by two authors (S.V. and C.L.), and discrepancies were resolved by consensus. For each category, quality ratings were assessed as either ‘good’ (score of 40), ‘fair’ (30), ‘poor’ (20) or ‘very poor’ (10), demonstrating the strengths and weaknesses within each study (Hawker et al., 2002). A total score was calculated to illustrate overall quality, ranging from 90 to 360, where higher scores indicate higher quality.

## Results

**Figure 1.**
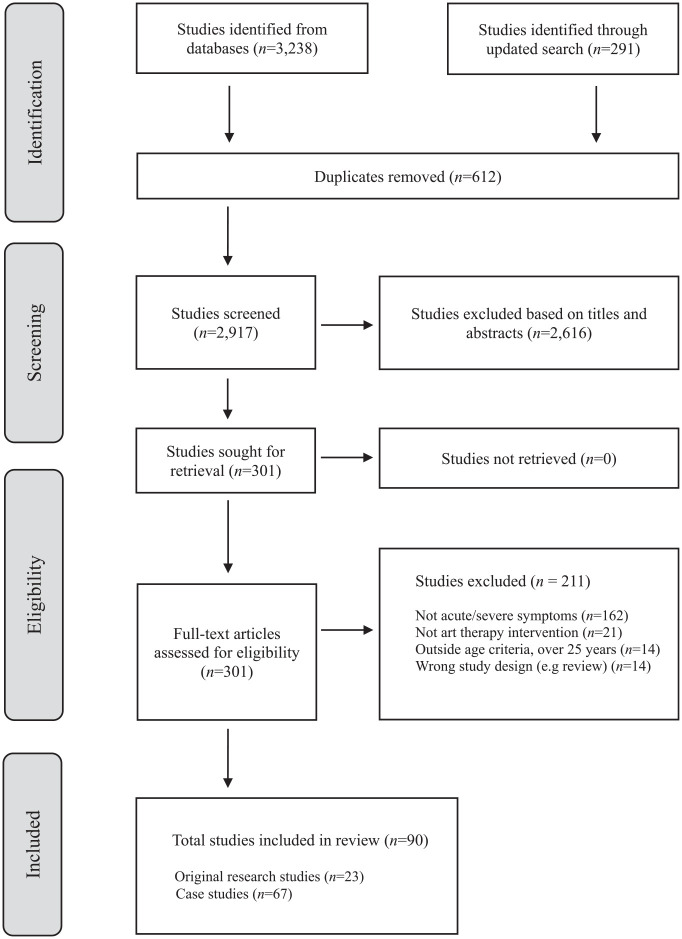
PRISMA flow diagram (Page et al., 2021).

A total of 3529 articles were extracted from all databases (Figure 1). After removal of 612 duplicates, and screening against inclusion and exclusion criteria, a total of 90 studies were included in the final synthesis. This comprised 67 case studies and 23 original research studies.

### Original research studies

Of 23 original research studies reviewed (Table 1), 9 took place in hospital-based mental health care settings. Most of these were inpatient child and adolescent psychiatric units (*n* = 7), in addition to a residential rehabilitation centre (*n* = 1), and a youth partial hospitalisation unit supporting transition out of hospital-based psychiatric care (*n* = 1). Nine studies took place in school environments, including a school set up within camps for internally displaced people, a post-psychiatric hospitalisation boarding school and an alternative schooling environment for ‘seriously emotionally disturbed’ young people. Other settings included community mental health care (*n* = 2), juvenile justice (*n* = 2) and out-of-home care (*n* = 1).

**Table 1.** Effectiveness and acceptability of art therapy interventions.

Author (year), country	Setting	Participant demographic details	Participant mental health characteristics	Intervention description	Control group description	Analysis	Effectiveness of art therapy	Acceptability of art therapy
Anazor et al. (2023), Nigeria	School	400 participants aged 16–18 years, (Mean = 17 years), 56% female and 44% male. Randomly assigned to treatment (*n* = 200) and control conditions (*n* = 200).	Post-traumatic stress disorder (PTSD) symptoms due to abduction.	12-week group art therapy programme delivered through pre-recorded interactive television instruction. Facilitator and teacher present in classroom for support.	Waitlist control	Quantitative: International Trauma Questionnaire (ITQ) administered pre-, post- and 6-month follow-up.	67% reduction of PTSD symptoms for the intervention group, including steady improvement after 6 months.	No attrition from study reported.
Bourassa (2021), Canada	Youth partial hospitalisation unit	7 participants aged 15–18 years. 5 female presenting and 2 non-binary.	Various diagnoses including depression, anxiety and panic attacks.	Two half-days eco-art therapy retreat (group). Participants were invited to create free and directed artwork with and in nature. Artworks were displayed at a public exhibition.	N/A	Quantitative: Six-question purpose-designed feedback form. Qualitative: Phenomenological analysis of artist statements.	Participants shared that being in nature, and the freedom to create offered them a sense of calm and a way to slow down.	All participants unanimously agreed they would participate again if offered and endorsed high enjoyment ratings (averaging 5.8/7, 7 = highest).
Blomdahl and Goulias (2024), Sweden	Child and adolescent psychiatry units	9 participants aged 13–17 years. 1 male, and 8 females.	Severe depression	10 weekly 60-minute sessions of manual-based phenomenological art therapy for depression. Sessions included an introduction, mindfulness exercises, an art theme, reflection on the artwork and a conclusion.	N/A	Quantitative: Beck Depression Inventory (BDI-II) self-report form, the Client Satisfaction Scales (CSS), Revised Children’s Anxiety and Depression Scale (child and parent versions (RCADS-25 C and RCADS-25 P), KIDSCREEN-10 (children and parents’ versions), Education, Work and Social adjustment Scale (children and parents’ versions, EWSAS-C and EWSAS-P).	BDI-II results showed a statically significant decrease in depression. RCADS-C25D results also indicated a reduction of depressive symptoms post-post-treatment, but the difference was not statistically significant. The adolescents’ ratings regarding anxiety symptoms (RCADS-C25) also showed a trend towards decreasing.	The acceptability and feasibility of the treatment was determined by examining participants’ attendance, dropouts and cancellations. There were no cancellations in the study. All participants completed 10 therapy sessions. Furthermore, the adolescents and their parents were satisfied with the treatment based on results of CSS.
Coles and Harrison (2018), England	Community mental health (recovery team)	7 white British female participants aged 18–25 years.	Various diagnoses including emotionally unstable personality disorder, bipolar affective disorder, recurrent depressive disorder and anxiety.	Weekly 90-minute group art therapy sessions over 18 weeks. Engaged in reflective and expressive activities within the context of a museum setting.	N/A	Quantitative: UCL Museum Well-being Measure (Younger Adult), Psychological Outcome Profiles (PSYCHLOPS) measure, Rosenberg Self-Esteem Scale. Qualitative: Reflective interviews with participants.	Unanimous positive changes reported by all participants and care coordinators, such as becoming more able to express difficult emotions and feeling less anxious around others. PSYCHLOPS tool indicated substantial improvements in majority of participants’ problems, functioning and well-being indicated by positive change score. Overall trend for self-esteem scores suggested positive outcomes for the majority of participants.	Participants formed connections in alignment with goal of social inclusion. Some continued to communicate and meet up for several months after the group ended.
Collins et al. (2023), Canada	Secure care centre, youth justice programme	13 participants aged 12–19 years. 5 male, 7 female, 1 agender. Race is described as 62% white, 23% indigenous and 15% black.	High-risk behaviour, multigenerational trauma and chronic and complex mental health concerns.	Weekly 1-hour individual art therapy session over 12 weeks. First six sessions focused on establishing rapport, exploring various art media and encouraging self-expression. Remaining six sessions aimed to enhance communication, hope and emotional regulation, fostering resilience by interpreting life experiences.	N/A	Quantitative: Children’s Hope Scale (CHS), the Resilience Scale (RS-25) and the Bridge Drawing with Path (BDP) art-based assessment, client satisfaction survey.	Increase in levels of hope and resilience pre–post-test for both the Children’s Hope Scale (CHS) by 29% and the Resilience Scale (RS-25) by 16%.	Participants reported high satisfaction with the art therapy sessions. All participants strongly agreed or agreed that the art therapy session was a good fit for them, and that they looked forward to the session.
Ezeh et al. (2023), Nigeria	School	470 participants aged 10–18 years randomly assigned to art therapy (*n* = 117), dance therapy (*n* = 117) or control (*n* = 236) groups. 57% male, 43% female for treatment. Religion 52% Islam, 48% Christian.	PTSD due to abduction and spending >24 hours in captivity.	Twice-weekly 2-hour interactive media-based group art therapy or dance therapy sessions were delivered via pre-recorded video. 20 sessions over 10 weeks. Both art and dance therapy groups encouraged creative self-expression, and exploring feelings related to their experience of abduction.	No treatment. 52% male, 48% female. Religion 54% Islam, 46% Christian.	Quantitative: International Trauma Questionnaire (ITQ) as a measure of PTSD symptoms.	Significant drop in PTSD scores of participants in art therapy post-intervention and at 6-month follow-up. Control group did not report a significant drop at either timepoint. While both art therapy and dance therapy were effective in reducing PTSD symptoms, dance therapy showed a marginally greater reduction in scores compared with art therapy.	Very high (96%) return rate for the study including pre-test, post-test and 6-month follow-up. Very low (4%) drop out in the process of the study.
Gatta et al. (2014), Italy	Residential rehabilitation centre	9 participants 13–18 years old (Mean = 15.5 years), 5 male, 4 female.	Diagnosed with personality disorders in clusters A and B according to the DSM-IV-TR	Weekly 90-minute group art therapy (18 sessions). 6 months in total, over two separate periods (May–June and Sept–Dec 2012). Art therapy utilising drawing, painting and music components.	N/A	Quantitative: MacKenzie’s Group Climate Questionnaire (GCQ). Qualitative: Clinical observations by facilitators of group content and dynamics.	The comparison of GCQ scores between the first and second cycles showed a marked reduction in the degree of conflict, and increased levels of involvement and avoidance in the second cycle.	Overall mean group climate in GCQ identified over the course of the 18 sessions was 38.6/72 (72 = highest) lying in the ‘rather strongly positive’ range. Overall attendance across 18 sessions was 44%.
Gul and Irshad (2018), Pakistan	Schools within internally displaced people camps.	60 participants aged 10–15 years. Assigned to treatment group (*n* = 30) or control (*n* = 30). 50% male, 50% female in both the intervention and control groups.	PTSD indicated by high scores on the PTSD Evaluation Scale Children and Adolescent Questionnaire (PTSD CAQ).	Weekly individual art therapy sessions. 8 sessions delivered over roughly 2 months. Combination of art therapy and behaviour therapy techniques i.e. deep muscle relaxation technique or progressive muscular relaxation was used.	No treatment	Quantitative: PTSD Evaluation Scale Children and Adolescent Questionnaire (PTSD CAQ) Qualitative: Semi-structured Interviews to gather demographic information and clinical history.	Statistically significant drop in PTSD symptoms on PTSD CAQ post-intervention when compared to control. Symptoms of re-experiencing of traumatic events, avoidance of stimuli associated with traumatic events, and symptoms of increased arousal due to traumatic events decreased significantly in the intervention group. Control group showed little variation on pre–post-test scores.	No attrition from study reported.
Iyendo et al. (2023), Nigeria	School	470 participants aged 10–18 years. Randomly assigned to art therapy (*n* = 118), music therapy (*n* = 117) or control (*n* = 212) groups. Treatment groups (art and music) 46% male, 54% female. Religion 56% Islam, 31% Christian, 13% ‘other’.	PTSD due to being kidnapped and stayed >24 hours in captivity, as indicated by high scores on the International Trauma Questionnaire (ITQ).	6-week interactive television-based group art therapy intervention (with two in-class art therapists to provide support) involved sculpting, painting and sketching. Interactive television-based group music therapy intervention. Participants watched/listened to/made music for 40 minutes, Monday to Friday over a 6-week period.	Waitlist control. Control group was 46% male, 54% female. Religion 57% Islam, 29% Christian, 14% ‘other’.	Quantitative: International Trauma Questionnaire (ITQ) administered pre, post-intervention and at 3-month follow-up.	Art therapy was shown to significantly reduce PTSD symptoms by 63.1%. Art therapy was more effective in reducing PTSD symptoms when compared to music therapy. The effect of art therapy was sustained and further improved slightly at 3-month follow-up. There was no significant drop in PTSD scores for the control group.	High (92%) study completion rate and return rate for art therapy and music therapy treatment groups.
Kondracka (2014), Poland	Inpatient child and adolescent psychiatric unit	15 female participants aged 13–18 years.	Diagnosis of anorexia nervosa (AN) according to ICD-10.	8-session art therapy group programme with including painting, costume design, landscapes, clay and collage. Programme delivered weekly over 8 weeks.	N/A	Mixed method evaluation survey – Likert scale-rated questions, and open-ended questions.	Evaluation survey responses indicated that participants were satisfied with their participation in the workshops, and that they had enriched their knowledge of artistic work. Participants stated that the activities boosted self-esteem and improved communication between participants.	All participants also indicated that they were ‘satisfied’ (50%) or ‘very satisfied’ (50%) with the way the workshops were conducted. All participants indicated they felt ‘safe’ (60%) or ‘very safe’ (40%) during the workshops.
Kymissis et al. (1996), USA	Inpatient child and adolescent psychiatric unit	37 participants aged 13–17 years. Assigned to treatment group (*n* = 18) or control group (*n* = 19).	Variety of diagnoses including conduct disorder, oppositional- defiant disorder, depressive disorder, bipolar disorder and borderline personality disorder.	17 group art therapy sessions over 5 weeks, focusing on the Synallactic Collective Image Therapy (SCIT) approach. Participants were asked to draw a picture with specific art materials provided. Group members presented their drawings and voted on one for discussion. The creator of the image with most votes shared meaning of the work with group. Other members were then asked to offer their associations.	Freeform ‘discussion group’ with minimal direction from therapists.	Quantitative: Children’s Global Assessment Scale (CGAS) and the Inventory of Interpersonal Problems (IIP).	CGAS scores were significantly lower pre–post-treatment for both control and art therapy groups. The art therapy group showed greater improvement in CGAS scores overall, although not statistically significant. Improvements in assertiveness for the art therapy group were significant in the Inventory of Interpersonal Problems Scale (IIP).	Anecdotal data suggests that the patients enjoyed the SCIT group, and that using artwork did facilitate the group process.
Liu et al. (2023), Nigeria	School	470 female participants aged 16–18 years (Mean = 17). Randomly assigned to art therapy group (*n* = 118), music therapy group (*n* = 117) or control group (*n* = 235)	Suicidal ideation indicated by the Suicide Ideation Questionnaire (SIQ-JR), in the context of surviving abduction. Participants had been captive for >24 hours involving molestation, rape and other hardship.	Interactive television-based art therapy or music therapy groups. The music therapy group involved watching, listening, and creating their own music. The art therapy group involved painting, sculpting and sketching. Art therapy sessions took place 1-hour daily from Monday to Friday over 6 weeks. Two therapists were present in classroom for support for both treatment groups.	Waitlist control	Quantitative: Suicide Ideation Questionnaire (SIQ-JR).	At baseline, all participants had high and similar suicide ideation scores. Those exposed to art and music therapies showed a significant reduction in suicidal ideation scores pre–post-intervention; however, control groups scores stayed the same. Those exposed to the art therapy intervention reported lower scores for suicidal ideation compared to the music therapy group, including at 3 months post-intervention.	High study completion rate (92%) for art therapy and music therapy treatment groups.
Lyshak-stelzer et al. (2007), USA	Inpatient child and adolescent psychiatric unit	29 participants aged 13–17 years (Mean = 15.07), randomised to treatment (*n* = 14) or control (*n* = 15) groups. Treatment group was 55% male, 45% female. Ethnicity was 40% African American, 35% Latino/a, 18% White, 0.7% Caribbean American, 5% mixed ethnicity, 0.7% Bangladeshi.	High levels of PTSD symptoms. Most participants reported exposure to multiple trauma events.	16 weekly 1-hour Trauma-focused Art Therapy (TF-ART) groups. Artworks from 13 directive sessions were compiled in a hand-made book format to express a narrative of the participant’s ‘life story’. 2 additional sessions were provided to organise and make front/back covers to the book. 1 session was for group members to share the final book with peers.	Art and craft activities group	Quantitative: The UCLA PTSD Reaction Index for DSM-IV, Child Version.	There was a significant effect of treatment over time for both groups across treatment conditions. There was a significant treatment by condition interaction showing that TF-ART was significantly more effective in reducing trauma symptoms from pre–post-treatment than the control group. There was also less incidents of restrictive practices used with participants in the treatment group.	Attrition was relatively high because patients were discharged prematurely relative to completing treatment protocols.
Nielsen et al. (2019), Australia	Inpatient child and adolescent psychiatric unit	46 participants aged 12–18 years.	Participants had severe mental illness (such as depression or schizophrenia) and had not recovered during an acute admission, requiring long-stay inpatient care.	Long-term group and individual art psychotherapy with optional verbal reflection. Questionnaires gathered over 7-year period, during which participants stayed generally 3 – 6 months in inpatient care.	N/A	Quantitative: Patient Satisfaction Questionnaire Qualitative: Analysis of artworks. Images were sorted by emerging categories, guided by visual grounded theory methodology.	78% of patients reported that art therapy helped them understand how their thoughts related to their feelings, 80% reported it helped them learn how to express themselves and 66% reported that it helped them develop self-confidence. Artworks were observed to change across the course of treatment to reflect the adolescent’s capacity to better tolerate their experiences of distress.	All participants rated the experience as either ‘excellent’ (37%), ‘great’ (37%) or ‘good’ (26%). Many expressed a desire to continue art therapy after leaving hospital.
Parkinson and Whiter (2016), United Kingdom	Early intervention psychosis service, Child and Adolescent Mental Health Services (CAMHS).	Participants aged 14 and over.	First episode psychosis	2-hour art therapy group including a short warm up exercise and reflection of the artwork, followed by a longer period of art-making, usually free of direction and ending with a longer period of talking and looking together at the work.	N/A	Qualitative: Two participants made audio image recordings	Participants reported benefits in self-confidence, changing perceptions about themselves and others, and managing difficult feelings through art-making. Art therapy helped them organise their thoughts and achieve a sense of accomplishment.	Art therapy accommodated different paces and ways of engagement. The non-intrusive nature of art therapy was highlighted by participants as a positive aspect.
Persons (2009), USA	Psychiatric unit within a juvenile correctional centre	46 male participants aged 16–20 years. Race was described as 52% African American and 48% Caucasian.	The most frequent diagnoses were major depressive disorder, PTSD, borderline personality disorder and conduct disorder. Most boys had also been diagnosed with attention deficit hyperactivity disorder.	Long-term group and individual art therapy between 2 months and 2 years (Mean = 8 months). Participants received from 2 to 10 hours (Mean = 5 hours) of individual and/or group art therapy per week. Non-directive therapeutic approach, however in individual therapy participants’ artworks would be processed, usually with experiential or gestalt procedures. Artworks were exhibited internally and outside of the facility.	N/A	Qualitative: Content analysis of transcribed interviews and thematic content analysis of artworks.	Participants reported that art therapy was beneficial in relieving stress, reducing boredom, increasing self-confidence and improving concentration. 85% reported learning to reduce their anger. 80% reported reducing their self-injurious behaviour and improving their relationships. 75% reported a reduction in depression. 65% reported reducing the amount of trouble they got into at the institution. 55% reported a reduction in anxiety and feeling more in control. 50% reported having more tolerance and acceptance.	Participation was voluntary. Participants were eager to participate in the programme, often doing extra work by painting in their rooms at night. According to participants, the positive recognition and encouragement they received in the programme were noted as important factors contributing to their engagement and enjoyment.
Pretorius & Pfeifer (2010), South Africa	Children’s home (Out of home care)	25 female participants aged 8–11 years (Mean = 9.6 years). Assigned to treatment (*n* = 13) or control groups (*n* = 12). Ethnicity was described as 6 black African girls, 2 coloured girls and 17 white girls.	Clinically significant trauma symptomatology as rated by the Trauma Symptom checklist for Children (TSCC), in the context of a history of sexual abuse.	8-session group art therapy programme based on existential humanistic perspective, and incorporating principles from Gestalt therapy, the client-centred approach and the abuse-focused approach.	Waitlist control	Quantitative: Trauma Symptom Checklist for Children (TSCC) and Human Figure Drawing (HFD).	The art therapy groups demonstrated significant reduction in depression, sexual trauma (as measured by TSCC) and anxiety (as measured by HFD) compared to the control groups. No change in levels of self-esteem were found (as measured by TSCC).	No attrition from study reported.
Tibbetts and Stone (1990), USA	Office of Education Special Class Alternative (SCA) setting.	20 participants aged 14–16 years assigned to treatment (*n* = 10) or control (*n* = 10) groups.	Participants deemed to have ‘Serious Emotional Disturbance (SED)’ signified by various mental health challenges relating to attention, interpersonal relationships, behaviour, mood and/or somatic symptoms.	6 weekly individual art therapy sessions, 45 minutes each in duration. Non-directive art therapy approach, informed by principals of Gestalt therapy.	Engaged in playing board games, talking about weekend activities and taking walks around the school grounds.	Quantitative: Roberts Apperception Test (RATC) and Burks Behaviour Rating Scale (BBRS)	While both groups demonstrated change overall, the art therapy treatment group showed significant reductions in various emotional measures, including Depression, Anxiety, Reliance Upon Others and Rejection (as measured by RATC). There were also significant improvements in Attention Span and Sense of Identity for the art therapy group (as measured by BBRS).	4 participants did not complete the study (2 from treatment, 2 from control group) for reasons that included expulsion, running away from home and attempted suicide.
Versitano et al. (2024a), Australia	Inpatient child and adolescent psychiatric unit	37 participants aged 6–16 years (Mean = 14.4 years). 10% male, 90% female.	Primary diagnosis: Mood disorders 26%, anxiety disorders 20%, eating disorders 15%, suicidal ideation 17%, overdose 10%, behavioural and emotional disorders 6%, schizophrenia, schizotypal or psychotic disorders 3%, other 3%.	Multidisciplinary therapeutic group programme delivered weekly over a 4-month period including art therapy, allied-health led therapy group, arts and crafts, ‘creative activities and games’ group, psychiatry-led therapy group, patient-led community feedback meeting, cooking and music.	N/A	Mixed method programme evaluation survey – calculation of closed questions, and content analysis of open-ended questions.	The art therapy group was reported to be the most helpful group (46%) by a substantial margin compared to all other groups offered in the programme.	Art therapy was one of the most consistently attended groups (89%) within the programme, and also reported as one of the most enjoyable (49%) by young people. Young people also suggested increased art therapy services on the unit.
Versitano et al. (2024b), Australia	Inpatient child and adolescent psychiatric unit	948 participants aged 6–18 years (Mean = 14.1 years), 80% female, 20% male.	A broad range of primary diagnoses, predominantly mood (affective) disorders (26%), which comprised primarily of major depressive disorders (21%). Young people were also admitted due to suicidal ideation (14%), overdose (11%) and anxiety disorders (10%).	The art therapy service included both group and individual art therapy interventions, integrating directive and non-directive approaches to art therapy, delivered by a masters-qualified art therapist.	N/A	Quantitative: Retrospective analysis of incidents of restrictive practices (seclusion, restraint, intramuscular injected sedation and PRN use or absconding in context of episode of seclusion or restraint). Comparing phases with art therapy service provision (38 months), to phases where there was no art therapy service (36 months).	Findings indicated a clear association between the provision of art therapy, and a statistically significant reduction in the prevalence of restrictive practices such as seclusion, physical restraint and intramuscular injected sedation on an acute inpatient psychiatric unit. These results indicate lower levels of acute distress for young people during times when art therapy is provided on the unit. Findings also demonstrated a significant reduction in length of stay and readmission rates.	N/R
Wolk and Bat Or (2023), Israel	Post-psychiatric hospitalisation boarding school	13 female participants aged 16–18 years (Mean = 17.4 years).	Various diagnoses including depression, anxiety, eating disorders, personality disorders, PTSD, adjustment disorder, suicidal ideation, self-harm, attention deficit hyperactivity disorder and learning disabilities.	Weekly 1-hour ‘open studio’ art therapy group offered over a 2-year period. Focus on embroidery. Each session started with inviting participants to express themselves using thread and embroider freely on a shared cloth. After that, each participant worked on their own personal project. At the end of each session, sharing and reflection on embroidery occurred.	N/A	Qualitative: Phenomenological Youth Participating Action Research method. Data analyses of interviews, focus groups and the observation of embroidery pieces together with the participants.	Findings suggest that embroidery had therapeutic benefits for this population and supports psychological development. This study reveals that embroidery, whose threads are intricately embedded in society and culture, and may provide a unique and meaningful activity for young people and enables a social and cultural exploration of self and community.	Long-term engagement with open studio art therapy embroidery groups over 2-year period.
Yi et al. (2024) Nigeria	School	450 participants aged 10–18 years (47% male, 53% female) were randomly assigned into control (*n* = 225) and treatment (*n* = 225). The treatment group was randomly divided into cognitive behaviour, art or music therapy (*n* = 75 per group).	High depression symptoms in the context of surviving abduction.	Each therapy (cognitive behavioural, art or music) had two weekly sessions (Mondays and Fridays) for 6 weeks, totalling 12 sessions. A session took 1 hour to complete; 10–15 min introduction, 35–45 min intervention and 5–10 min conclusion/closing. Sessions were delivered as interactive television-based recordings, with in-class support.	Waitlist control	Quantitative: Children Depression Inventory (CDI) administered pre-, post- and 3-month follow-up.	Art, music and cognitive behaviour therapies delivered through interactive television contributed 73% to reducing depression symptoms. CBT was deemed most effective, closely followed by art therapy, and then music therapy including at 3-month follow-up.	96% return rate for the treatment group survey responses.
Zhang et al. (2023), Nigeria	School	470 participants aged 10–18 years randomly assigned to treatment groups of either art therapy (*n* = 78), music therapy (*n* = 78), poetry therapy (*n* = 79) or a control group (*n* = 235). Intervention groups comprised 51% males, 49% females. Religion was 52% Islam, 28% Christianity, 20% ‘Other’.	PTSD due to being kidnapped and stayed >24 hours in captivity, as indicated by high scores on the International Trauma Questionnaire (ITQ).	6 weeks of interactive television-based art therapy, music therapy or poetry therapy was delivered. One-hour sessions took place 5 days a week Monday to Friday.	Waitlist Control	Quantitative: International Trauma Questionnaire (ITQ) administered pre-, post- and 3-month follow-up.	Results showed significant main effect of treatment conditions in the reduction of PTSD symptoms. Intervention delivered through interactive television contributed to a 63% decrease in PTSD symptoms compared to the natural decrease measured in the control group. Those exposed to art therapy showed more decline in PTSD symptoms than music therapy and poetry therapy in lowering PTSD symptoms. A similar result was sustained even at 3-month follow-up.	No attrition reported from this study. 100% completion of survey responses reported.

N/R: Not reported; N/A: Not applicable.

### Participant demographics

There were a total of 4053 participants within 23 studies. Of these, 1400 were male, 2508 were female, 3 participants were non-binary or agender and 142 participants were uncategorised. Participants presented with a range of severe and/or acute mental health challenges. Seven studies focused specifically on young people with PTSD or severe post-traumatic stress symptoms. Ten studies included participants with various mental health conditions including mood disorders, personality disorders, anxiety disorders, eating disorders, conduct disorders, schizophrenia or psychotic spectrum disorders, or neurodevelopmental disorders (co-occurring with a mental health condition). The remaining studies focused on a single diagnosis or presentation, including severe depression, anorexia nervosa, first episode psychosis, personality disorders and suicidal ideation. Studies were conducted across diverse geographic locations, predominantly Africa (*n* = 7), Europe (*n* = 5), the United States (*n* = 4) and Australia (*n* = 3).

### Art therapy interventions

The art therapy interventions offered varied substantially. Most interventionists were art therapists (*n* = 18) with minor variation in terminology such as ‘art psychotherapist’, ‘certified art therapist’, ‘qualified art therapist’, ‘registered art therapist’ or ‘professional art therapist’. Five studies reported on art therapy interventions facilitated by other health professionals such as psychologists, psychiatrists or psychotherapists.

Most studies delivered art therapy interventions in a group format (*n* = 16). Four focused specifically on individual art therapy sessions, while three provided both group and individual interventions. Study duration ranged from two half-day workshops, up to 2 years of art therapy service delivery. The length of participant engagement varied, and often depended upon their length of stay, particularly within inpatient and juvenile justice settings. Session frequency varied from one to five times per week, and sessions ranged from 45 minutes, to a half day in duration. Longer sessions were often group-based, while briefer interventions tended to be individual sessions. Some studies utilised an open studio approach (*n* = 4), wherein participants could come and go from the artmaking space with the art therapist always present to provide support (Finkel and Bat Or, 2020).

Intervention structure also varied. Many interventions could broadly be categorised as either directive (*n* = 11) – guided by specific themes or structured activities – or non-directive (*n* = 6) – client-led exploration of organically emerging themes or ideas (Kottman and Dickinson, 2016). Some studies utilised a mixture of directive and non-directive components (*n* = 6), often structuring sessions responsively based on participant need. Art therapy practices can occur across a continuum, at times incorporating guiding elements, as well as opportunities for self-directed exploration (Kottman and Dickinson, 2016). Intervention descriptions are outlined in Table 1.

### Control groups

Eleven studies utilised a control group, five of which were waitlist control and two which received no treatment. Of the remaining three control groups, one was an arts-and-crafts activities group (Lyshak-Stelzer et al., 2007), another was a freeform discussion group (Kymissis et al., 1996). The other involved a mixture of recreational activities and conversation (Tibbetts and Stone, 1990).

### Effectiveness of art therapy

With substantial variation in research design and methodology, effectiveness was challenging to assess across the original research studies. The most definitive measure of effectiveness was the reduction of PTSD symptoms, as demonstrated across six studies. Most studies measured PTSD symptom severity using the International Trauma Questionnaire (Anazor et al., 2023; Ezeh et al., 2023; Iyendo et al., 2023; Zhang et al., 2023), while other studies used the UCLA PTSD Index (Lyshak-Stelzer et al., 2007) or PTSD Evaluation Scale Children and Adolescent Questionnaire (Gul and Irshad, 2018). Art therapy was effective in significantly reducing PTSD symptom severity for young people across all six studies (Anazor et al., 2023; Ezeh et al., 2023; Gul and Irshad, 2018; Iyendo et al., 2023; Lyshak-Stelzer et al., 2007; Zhang et al., 2023). Gains were maintained at 3 months (Iyendo et al., 2023; Zhang et al., 2023) and 6 months post-intervention (Ezeh at al., 2023) in follow-up studies. One study demonstrated that while both trauma-focused art therapy and an arts-and-crafts control group reduced PTSD symptoms (Lyshak-Stelzer et al., 2007), the art therapy intervention was comparatively more effective. Another study also found that the art therapy intervention was associated with a reduction in trauma symptoms related to sexual abuse history, as compared to the control (Pretorius and Pfeifer, 2010). One study showed that a dance therapy group and an art therapy group were both significantly effective in reducing PTSD symptoms compared to the control, but that dance therapy was marginally more effective (Ezeh et al., 2023). However, another study (Iyendo et al., 2023) demonstrated that art therapy was more effective in reducing PTSD symptoms compared to music therapy and poetry therapy interventions. Art therapy was also more effective than music therapy in reducing suicidal ideation (Liu et al., 2023) and depression (Yi et al., 2024).

Studies also reported improved emotion regulation, with participants managing difficult emotions through the artmaking process (Parkinson and Whiter, 2016) and feeling a greater sense of calm (Bourassa, 2021). Another study within a juvenile justice setting reported that participants who undertook the art therapy intervention had reduced anger, felt more in control and were getting into less ‘trouble’ (Persons, 2009). One study demonstrated a significant association between art therapy interventions and a reduction in seclusion, restraint and intramuscular injected sedation on an acute inpatient child and adolescent mental health unit (Versitano et al., 2024b). Improved emotional regulation and distress tolerance were further evidenced by a significant reduction in suicidal ideation (Liu et al., 2023) and a reduction in self-injurious behaviour (Persons, 2009). Cognitive improvements and regulation were also reported in relation to improved concentration (Persons, 2009), attention span (Tibbetts and Stone, 1990) and organisation of thoughts (Parkinson and Whiter, 2016).

Synthesis of findings across the remaining studies revealed several key themes. Studies demonstrated effectiveness of art therapy interventions in improving self-efficacy and empowerment, demonstrated by increased levels of self-confidence (Nielsen et al., 2019; Parkinson and Whiter, 2016; Persons, 2009), assertiveness (Kymissis et al., 1996) accomplishment (Parkinson and Whiter, 2016) and self-esteem (Coles and Harrison, 2018; Kondracka, 2014).

Art therapy interventions were also effective in increasing self-awareness (Parkinson and Whiter, 2016; Wolk and Bat Or, 2023), helping young people to understand how their thoughts related to their feelings (Nielsen et al., 2019) and improving their sense of identity (Tibbetts and Stone, 1990). Art therapy also improved self-expression (Coles and Harrison, 2018; Nielsen et al., 2019) and increased hope and resilience (Collins et al., 2023).

Two studies identified significant reductions in depressive symptoms for young people engaging in art therapy (Blomdahl and Goulias, 2024; Yi et al., 2024). While art therapy, music therapy and cognitive behaviour therapy (CBT) were all significantly effective in reducing depression (Yi et al., 2024), CBT was deemed most effective, followed by art therapy and finally music therapy. At 3 months post-intervention, CBT and art therapy groups demonstrated similar outcomes, sustaining a significant reduction in depression scores when compared to the control (Yi et al., 2024). A further three studies reported lower levels of depression and anxiety (Persons, 2009; Pretorius and Pfeifer, 2010; Tibbetts and Stone, 1990). Studies also demonstrated that art therapy enhanced overall well-being (Coles and Harrison, 2018; Kymissis et al., 1996; Nielsen et al., 2019).

Improvements in interpersonal effectiveness were demonstrated by improved communication (Kondracka, 2014), reduction in group conflict (Gatta et al., 2014) and participants feeling less anxious around others (Coles and Harrison, 2018). There were also reported improvements in participants’ relationships with others, including feeling more tolerance and acceptance (Persons, 2009).

While predominantly positive outcomes were reported, one study found an increase in avoidance within the group climate post-intervention (Gatta et al., 2014). However, authors indicated this avoidance appeared to enhance group cohesion, providing greater structure to participation and reducing conflict (Gatta et al., 2014).

### Acceptability of art therapy

Acceptability of art therapy interventions was demonstrated in the majority of original research studies (*n* = 20/23), though the quality of evidence varied. Survey responses gathering direct feedback from young people indicated high enjoyment and satisfaction ratings for art therapy interventions across several studies (Blomdahl and Goulias, 2024; Bourassa, 2021; Collins et al., 2023; Kondracka, 2014; Nielsen et al., 2019; Versitano et al., 2024a). Within a multidisciplinary group programme on an acute inpatient unit, art therapy was one of the most consistently attended and enjoyed groups, compared to other therapeutic and diversional interventions offered (Versitano et al., 2024a). Young people also requested increased art therapy services (Versitano et al., 2024a). Similarly, within a long-stay inpatient setting, participants unanimously indicated their experience of art therapy was positive, rating it as excellent (37%), great (37%) or good (26%), with many young people wanting to continue art therapy post-discharge (Nielsen et al., 2019). Within an inpatient mental health setting for young people diagnosed with anorexia nervosa (Kondracka, 2014), participants had a unanimously positive response to the intervention, reporting being ‘satisfied’ (50%) or ‘very satisfied’ (50%), and feeling ‘safe’ (60%) or ‘very safe’ (40%), in art therapy workshops. In a youth justice programme (Collins et al., 2023), all participants reported high intervention satisfaction ‘strongly agreeing’ or ‘agreeing’ that art therapy sessions were a good fit for them and that they looked forward to sessions (Collins et al., 2023). In a study utilising manual-based phenomenological art therapy for depression (Blomdahl and Goulias, 2024), acceptability was indicated by no cancellations or programme dropouts and a high overall client satisfaction score.

Qualitative data also demonstrated acceptability of the art therapy interventions. In one study undertaken in a psychiatric unit within a juvenile correctional centre (Persons, 2009), participants shared that receiving positive recognition and encouragement in the art therapy programme were important factors contributing to their engagement and enjoyment. Furthermore, these young people were eager to participate in this voluntary art therapy programme, often continuing their artworks in their rooms at night, and spending up to 10 hours per week in group and/or individual art therapy sessions (Persons, 2009). Another study exploring a museum-based art therapy intervention (Coles and Harrison, 2018) found that participants formed positive social connections, and some continued to meet as a group several months post-intervention. Participants also stated the non-intrusive nature of art therapy accommodated for different paces and ways of engagement in an early intervention psychosis programme (Parkinson and Whiter, 2016).

Where studies made no specific reference to intervention acceptability, attrition was considered as a proxy indicator. Three studies reported no attrition (Anazor et al., 2023; Gul and Irshad, 2018; Pretorius and Pfeifer, 2010), while a further three reported very high study completion rates (all >92%) (Ezeh at al., 2023; Iyendo et al., 2023; Liu et al., 2023; Yi et al., 2024). However, one study of an inpatient mental health setting mentioned relatively high attrition due to patients being discharged prior to completing the 16-session treatment protocol (Lyshak-Stelzer et al., 2007). Another study indicated an 80% study completion rate in an alternative education environment, due to participant expulsion, absconding or a suicide attempt (Tibbetts and Stone, 1990). One study reported 44% attendance to a group art therapy programme offered in an inpatient mental health unit (Gatta et al., 2014). However, authors described attendance patterns as typical of the stability and continuity of participant engagement in that particular setting. When exploring anything participants did not like about art therapy workshops offered in an inpatient psychiatric setting (Kondracka, 2014), criticism of verbal components within the workshops was raised by some participants.

### Quality assessment of included studies

Quality assessment details are included in Table 2. Almost all studies demonstrated ‘fair’ to ‘good’ introduction, method and results sections. Overall, randomised controlled trials (RCTs) exhibited strengths in areas of methodology, data analysis and results, with some studies achieving consistently high ratings across all assessed components, such as methodological rigour, ethical consideration and generalisability of findings (Ezeh et al., 2023; Yi et al., 2024). However, certain RCTs received lower scores in areas of ethics, bias and sampling, exposing potential limitations in their methodological execution and sample representativeness (Kymissis et al., 1996; Tibbetts and Stone, 1990). Although sampling details regarding age, gender, race and context were generally adequate, sample size emerged as a recurrent limitation, likely reducing generalisability of findings and diminishing statistical power. It is noteworthy that the assessment tool used (Hawker et al., 2002) did not allow for a comprehensive evaluation of sample size. Several cohort and cross-sectional studies exhibited weaknesses in sampling, ethical considerations and data analysis, affecting the reliability and generalisability of their results.

**Table 2.** Quality assessment of original research studies.

	Abstract and title	Intro and aims	Method and data	Sampling	Data analysis	Ethics and bias	Findings/results	Transferability/generalisability	Implications and usefulness	Overall rating (/360)
**Randomised control trial**										
Lyshak-stelzer et al. (2007)	40	40	40	40	30	30	40	30	30	320
Zhang et al. (2023)	40	40	30	40	40	30	40	30	40	320
Kymissis at al. (1996)	20	30	30	30	40	20	40	30	30	270
Tibbetts and Stone (1990)	20	30	30	30	40	20	40	30	40	280
Ezeh et al. (2023)	40	30	40	40	40	40	40	40	40	350
Yi et al. (2024)	40	40	40	40	40	40	30	40	40	350
**Quasi-experimental**										
Anazor et al. (2023)	30	40	30	30	30	30	40	30	30	290
Gul and Irshad (2018)	30	30	40	30	20	20	40	30	30	270
Pretorius and Pfeifer (2010)	30	30	40	30	30	20	40	40	30	290
Parkinson and Whiter (2016)	30	30	30	30	30	20	30	30	30	260
Iyendo et al. (2023)	40	40	40	40	40	40	40	40	40	360
Persons (2009)	40	40	40	40	40	30	40	40	40	350
Liu et al. (2023)	40	40	40	40	40	40	40	40	40	360
Blomdahl and Goulias (2024)	40	40	40	30	40	40	40	40	40	350
**Cohort**										
Gatta et al. (2014)	20	30	30	20	30	10	30	20	30	220
Wolk and Bat Or (2023)	40	40	40	30	40	40	40	30	30	330
Bourassa (2021)	40	30	30	20	30	30	30	20	30	260
Kondracka (2014)	20	20	30	20	20	10	20	20	20	180
Versitano et al. (2024a)	40	40	30	40	30	40	40	40	40	340
**Cross-sectional**										
Versitano et al. (2024b)	40	40	30	30	30	30	40	30	40	310
Nielsen et al. (2019)	30	30	30	20	30	30	40	30	30	270

### Case studies

Of 90 identified eligible studies, the remaining 67 used case study methodology to examine art therapy interventions (Supplementary Table 1). Similar to the original research studies, most took place within inpatient child and adolescent psychiatric units (*n* = 34), in addition to outpatient community mental health settings (*n* = 14), residential treatment centres (*n* = 4), schools (*n* = 4), juvenile justice and forensic facilities (*n* = 2), private practice (*n* = 2) and specially designed programmes or camps for young people (*n* = 2).

Case study methodology most often entailed descriptive analysis of a single young person (*n* = 35) or multiple case study participants (*n* = 19), as compared with a group analysis (*n* = 13). Most studies (*n* = 45) focused on young people with comorbid mental health conditions, such as adjustment disorder, mood and anxiety disorders, eating disorders, conduct and behavioural disorders, personality disorders, schizophrenia or psychotic spectrum disorders, and neurodevelopmental disorders (co-occurring with a mental health condition). Fewer case studies focused on a single diagnosis or presentation (*n* = 22), most commonly conduct or behavioural disorders (*n* = 5) or developmental trauma (*n* = 5).

Art therapy interventions again varied substantially in type, format and duration. Most interventions were delivered on an individual basis (*n* = 42). Theoretical frameworks or guiding principles were unclear or unspecified in many interventions. Overall, older studies utilised predominantly psychoanalytic or psychodynamic approaches to intervention and interpretation (e.g. Allie, 1981; Naumburg, 1945). More recent studies have continued to utilise psychodynamic approaches to art therapy (e.g. Hanvey and Tepper-Lewis, 2019), alongside more modern and alternative theoretical frameworks including attachment-informed art therapy (Cunningham and Page, 2001; Kozlowska and Hanney, 2001), ecopsychology (Wardle, 2023), land art (Bourry and Barbe, 2012), somatic experiencing (Hetherington and Gentile, 2022) and trauma processing methods (Howie et al., 2002; Mallay, 2002), as well as transcultural family therapy (Roijen, 1991).

Most interventions took a multidisciplinary and multimodal intervention approach, particularly within hospital-based settings. Art therapy interventions were frequently delivered by a trained art therapist alongside other health professionals including psychiatry, psychology, social work, case management intervention and educational therapy (e.g. Kozlowska and Hanney, 2001; McGann, 1999; Rupa et al., 2014). Provision of art therapy often took place in conjunction with family-based treatments, medication management and talk-based psychotherapies such as CBT (Coşkunlu et al., 2018; Kozlowska and Hanney, 2001; Mazloomian and Moon, 2007). Some interventions also offered additional treatment components such as bullying intervention (e.g. Buchan, 2009), behaviour management strategies (Hetherington and Gentile, 2022; Potash, 2009) and other creative therapies such as music therapy, play therapy and psychodrama (Diamond-Raab and Orrell-Valente, 2002; Mazloomian and Moon, 2007; Sikes and Kuhnley, 1984). Session frequency ranged from 3 to 4 times per week to monthly, with session duration from 30 to 90 minutes. The longest case study was conducted over a 3-year period (Steinhardt, 1995).

Outcome measures were heterogeneous, precluding clear synthesis. Qualitative measures were most commonly used, involving clinical observations undertaken by the art therapy interventionist. Only three case studies used validated qualitative coding methods or frameworks; all of which used a phenomenological coding approach focused on identifying themes in participant artwork, verbal dialogue and semi-structured interviews (Bourry and Barbe, 2012; Van Lith, 2008; Wyder, 2019). Four studies used art-based assessment measures to examine efficacy of art therapy. This included the Levels of Emotional Awareness Scale (Barth and Klosinski, 2007) to measure emotional awareness, an Observation Grid to identify mental activity and pictorial mediation in childhood psychosis (Calestrémé et al., 2016), the Body Image Projective Drawing Test to assess unconscious internal processes (Kim and Ki, 2014) and the Face Stimulus Assessment Task, a projective drawing assessment (Robb, 2002). Quantitative methodologies were rarely used to evaluate outcomes of art therapy interventions. Only four case studies used validated questionnaires to assess self-reported mental health outcomes, all of which reported benefits of art therapy. This included reductions in depressive symptom severity (Child Depression Inventory, Coşkunlu et al., 2018), post-traumatic stress symptoms (Impact of Events Scale-Revised, Wyder, 2019), alexithymia and somatisation symptoms (Alexithymia Scale Questionnaire and Symptom Checklist Revised, Kim and Ki, 2014) and improved self-esteem (School Form of Coopersmith Self-Esteem Inventory, Stanley and Miller, 1993).

Overall, outcomes were largely positive across the included case studies. Positive gains were reported in 54 of 67 studies, including improvements in emotional awareness and expression, interpersonal skills, cognitive skills and occupational functioning. Improvements in mental health outcomes were also reported, including most commonly reductions in post-traumatic stress symptoms (Harnden et al., 2004; Hetherington and Gentile, 2022; Kozlowska and Hanney, 2001; Peake, 1987; Potash, 2009; Stanley and Miller, 1993; Waller, 2006; Wardle, 2023). Reductions were also observed in symptoms of anxiety (e.g. Buchan, 2009; Calestrémé et al., 2016), disordered eating (Diamond-Raab and Orrell-Valente, 2002; Lindinger and Karwautz, 2016), depression (Coşkunlu et al., 2018; Rupa et al., 2014), oppositional and behavioural difficulties (e.g. Fliegel, 2000; McGann, 1999), psychotic spectrum symptoms (e.g. Atlas et al., 1992; Calestrémé et al., 2016), somatisation symptoms (Kim and Ki, 2014), self-harming behaviour (e.g. Briks, 2007; Coşkunlu et al., 2018) and suicidal ideation and attempts (e.g. Coşkunlu et al., 2018; Diamond-Raab, Orrell-Valente, 2002). Unclear or mixed outcomes were reported in the remaining 13 studies across varied outcomes. Heterogeneity in intervention components, frameworks and intervention dosage, duration and format preclude distinction of particular components of art therapy contributing to effectiveness. Multimodal interventions also limit disentangling impacts of art therapy intervention from broader systemic or contextual treatments.

Around half the studies reported on acceptability (*n* = 34). Most indicated high acceptability and of positive impressions and experiences of art therapy (*n* = 24). This was inferred from clinical observations of participant engagement and enthusiasm, self-reports of high satisfaction and enjoyment, staff feedback on increased engagement (e.g. Barth and Klosinski, 2007), improved self-regulation (e, g, Coşkunlu et al., 2018), positive interaction with varied art materials (e.g. Howie et al., 2002) increased independent art-marking time (e.g. Wolf, 1975) and strong therapeutic rapport (e.g. Moon, 1999). Ten case studies reported negative or mixed acceptability, most commonly relating to poor participant engagement or participation (McGann, 1999), limited parental capacity in family-based art interventions (Nielsen et al., 2021; Roijen, 1991), limited motivation, resistance or premature termination of participation (George and Kasinathan, 2014; Hanvey and Tepper-Lewis, 2019; Horovitz, 1983; Powers and Langworthy, 1978; Raghuraman, 2000; Robb, 2002) or ‘therapy-interfering behaviours’ such as disrupting peers or engaging in conflictual behaviour (Bennink et al., 2003). Overall, this supports original research studies demonstrating generally positive impressions and engagement with art therapy interventions.

## Discussion

Clear patterns of effectiveness have emerged in art therapy interventions, despite variation in methodological approach and intervention structure. Multiple original research papers, predominantly randomised control trials, reported statistically significant reductions in PTSD symptom severity (e.g. Anazor et al., 2023; Ezeh et al., 2023; Zhang et al., 2023), with case study evidence mirroring this finding (e.g. Harnden et al., 2004; Hetherington and Gentile, 2022; Wardle, 2023). Studies also demonstrated effectiveness for emotional regulation and reducing acute distress. This was evidenced by reduced anger, greater emotional control (Persons, 2009), a statistically significant reduction in restrictive practices (Versitano et al., 2024b), as well as reductions in suicidal ideation (Liu et al., 2023), and self-injurious behaviour (Coşkunlu et al., 2018; Edan and Knecht-Favrod, 2011; Persons, 2009). Original research studies also indicated improved self-awareness (Parkinson and Whiter, 2016; Wolk and Bat Or, 2023), confidence (Nielsen et al., 2019; Parkinson and Whiter, 2016) and significantly reduced depression (Blomdahl and Goulias, 2024; Yi et al., 2024) and anxiety symptoms (Coles and Harrison, 2018; Pretorius and Pfeifer, 2010; Tibbetts and Stone, 1990).

Acceptability of art therapy interventions was consistently demonstrated, with high rates of attendance to art therapy sessions (Blomdahl and Goulias, 2024; Versitano et al., 2024a), and minimal attrition reported (e.g. Pretorius and Pfeifer, 2010; Yi et al., 2024). Many young people expressed enjoyment of art therapy interventions (e.g. Blomdahl and Goulias, 2024; Versitano et al., 2024a) and a preference for art therapy relative to verbal therapies (Diamond-Raab, Orrell-Valente, 2002; Gmitrowicz et al., 2012; Versitano et al., 2024a). Despite evidence predominantly supporting the acceptability of art therapy, some studies reported feasibility challenges regarding recruitment and retention in acute mental health settings where discharges were difficult to anticipate (Gatta et al., 2014; George and Kasinathan, 2014; Lyshak-Stelzer et al., 2007). Some case studies also reported challenges with participant engagement or motivation (e. g. McGann, 1999; Robb, 2002).

Art therapy was predominantly found to be effective in reducing symptom severity, while also being an enjoyable therapeutic modality with high levels of acceptability reported. These findings support those of a recent systematic review of the effectiveness of art psychotherapy for children and adolescents with mental health conditions (Braito et al., 2022) which found benefits for young people with PTSD symptoms. In addition, a scoping review of the use of visual arts in hospital environments, which included art therapy studies, found that arts and art therapy enhanced well-being and improved communication processes in health settings (Ullán and Belver, 2021). While both arts-and-health and art therapy interventions provide benefits, it is important to distinguish between the psychotherapeutic practice of art therapy and diversional arts-and-health activities, as they are frequently conflated, diminishing the strengths of each modality (Van Lith and Spooner, 2018).

Within the present systematic review, RCTs demonstrated that art therapy was a comparatively more beneficial intervention for young people than arts-and-crafts control groups (Lyshak-Stelzer et al., 2007), or freeform discussion control groups (Kymissis et al., 1996). This is particularly salient when working with young people experiencing severe or acute mental health challenges, as art therapists’ capacity to hold and contain complex, and often distressing, emotional and visual material in-session is supported by their specialised clinical training in the intersection of arts and psychotherapy (Rubin, 2011; Van Lith and Spooner, 2018). With 18 of the 23 original research studies conducted by trained art therapists, current findings are primarily applicable to art therapist-delivered treatments.

A systematic narrative review on art therapy for psychosocial problems in children and adolescents (Bosgraaf et al., 2020) found similar variations in art therapy interventions offered, as in our review. Bosgraaf et al. (2020) concluded that this represents a responsiveness of art therapists, and their capacity to flexibly adapt to the clinical needs and circumstances of young people, therefore providing positive outcomes. Art therapy is sometimes critiqued for its lack of manualised or structured (and therefore readily replicable) treatments. Art therapists are trained in client-focused work and deliver interventions tailored to specific client needs, requiring advanced therapeutic skills and sensitivities. This is a strength rather than deficit of the modality, consistent with evidence-informed practice that incorporates client perspectives and practice wisdom alongside scientific evidence (Alla and Joss, 2021; Psychotherapists and Counsellors Federation of Australia [PACFA], 2019).

Our review highlights the value of art therapy for young people experiencing severe or acute mental health conditions. Art therapy is sometimes misconceived as a diversional intervention or secondary treatment. However, current findings suggest it is an effective intervention which young people deem enjoyable and helpful. Therefore, art therapy services delivered by qualified art therapists could be established in more services which support young people who are experiencing severe or acute mental health challenges. This body of evidence is particularly strong in the treatment of PTSD symptoms. While this may not be possible in certain geographic locations due to workforce constraints, interactive media-based art therapy interventions have also demonstrated their effectiveness (e.g. Anazor et al., 2023; Liu et al., 2023; Zhang et al., 2023)

Several limitations warrant consideration. Non-English studies were included and translated using Google Translate which may affect the reliability of reported findings in those studies (Jackson et al., 2019). Furthermore, the review process included all forms of research including case studies. While qualitative research, particularly utilising case studies, has often been the preferred method of art therapy research historically, this poses challenges to generating evidence on effectiveness and acceptability, as cases are often selectively presented.

Future research on art therapy with young people experiencing severe and/or acute mental health conditions should aim to increase methodological rigour across the range of quantitative, mixed-method and qualitative approaches. This includes conducting further gold-standard evidence-based evaluations, particularly RCTs, which incorporate longitudinal follow-up assessments to discern intervention sustainability for a transdiagnostic cohort in acute mental health settings. Further research is also needed on the physiological effects of art therapy on mental health (Czamanski-Cohen and Weihs, 2016), examining potential mediators of effects including intervention structure, dose, delivery mode and outcomes assessed alongside confounding factors such as provision of multicomponent multidisciplinary interventions. A component analysis to identify the ‘effective ingredients’ of art therapy would provide further clarity. Future research may also examine condition-specific RCTs to strengthen evidence-based evaluations in areas with limited research to date, e.g., with obsessive compulsive disorder (Dyason et al., 2022). Future art therapy research should also ensure diversity representation, inclusivity and cultural sensitivity in its practice (Hughes et al., 2024), as this was under-reported or absent from many reviewed studies.

## Conclusion

Art therapy has significant therapeutic effects for young people who may be experiencing severe emotional distress. The novelty of this review lies in its specific focus on severe and acute mental health conditions for children and young people. This is pertinent given increasing trends in acute mental health presentations including self-harm and suicidal ideation within this population (Sara et al., 2023). This review identified effectiveness of art therapy in reducing symptoms of PTSD, depression, anxiety and suicidal ideation. Art therapy was also found to improve emotional regulation, confidence, self-awareness, distress tolerance, self-expression, interpersonal effectiveness, hope and resilience. Effectiveness across a range of clinical diagnoses, settings and art therapy intervention types reflects the responsive capacity of art therapy to adapt to the specific needs of young people it supports (Bosgraaf et al., 2020). Art therapy was a widely accepted intervention, with young people reporting high enjoyment, engagement and satisfaction with this therapeutic modality. Previous reviews have not examined acceptability (Braito et al., 2022; Cohen-Yatziv and Regev, 2019; Moula, 2020) despite this being a key consideration in the design, evaluation and implementation of healthcare interventions (Sekhon et al., 2017), further enhancing the value of this review and of art therapy intervention for young people. Art therapy services were found to be in settings which provide mental health care for young people with acute or severe mental health conditions, including hospitals, schools, juvenile justice and community mental health settings. This review highlights a strong and growing evidence base to support the provision of art therapy in the context of acute or severe mental health conditions in children and young people.

## Supplemental Material

sj-docx-1-anp-10.1177_00048674251361731 – Supplemental material for Art therapy with children and adolescents experiencing acute or severe mental health conditions: A systematic review Supplemental material, sj-docx-1-anp-10.1177_00048674251361731 for Art therapy with children and adolescents experiencing acute or severe mental health conditions: A systematic review by Sarah Versitano, Stephanie Tesson, Chae-Weon Lee, Sheridan Linnell and Iain Perkes in Australian & New Zealand Journal of Psychiatry
