# Supplementary material for: Art therapy with children and adolescents experiencing acute or severe mental health conditions: A systematic review
Source: Aust N Z J Psychiatry. 2025 Sep 8;59(10):863–87. doi: 10.1177/00048674251361731 (PMC12460915; doi:10.1177/00048674251361731)
Supplement: sj-docx-1-anp-10.1177_00048674251361731 – Supplemental material for Art therapy with children and adolescents experiencing acute or severe mental health conditions: A systematic review [file sj-docx-1-anp-10.1177_00048674251361731.docx]

| **Author (Year), Country** | **Setting** | **Participant demographic details** | **Participant mental health characteristics** | **Intervention description** | **Analysis** | **Effectiveness of art therapy** | **Acceptability of art therapy** |
| --- | --- | --- | --- | --- | --- | --- | --- |
| Allie (1981), N/R | N/R | 11-year-old male. | Schizophrenia. | Individual art therapy sessions. Intervention aimed to support emotional expression, build frustration tolerance and support identity development, consistent with a psychodynamic approach. Materials included drawing, body tracings, plasticine and play. | Qualitative: Clinical observations | Clinician reported that art therapy sharpened self-object differentiation and supported the child’s defensive system and greater autonomy. | N/R |
| Atlas et al. (1992), USA | Inpatient acute adolescent unit. | 4 participants aged 14-16 years. 1 male, 3 female. | Various diagnoses including psychosis, suicidal depression, and conduct disorder. | Individual art therapy. Intervention aimed to facilitate self-expression and reflection using poetry, drawing, and clay production. Delivered in tandem with supportive brief psychotherapy and medication reviews. | Qualitative: Clinical observations | Clinician reported that: - Participant 1 showed reduction in hallucination symptoms. - Participant 2 showed reduced preoccupation with death and increased verbalisation. - Participant 3 showed improved hopefulness, although mood swings and withdrawal persisted and fluctuated.  - Participant 4 showed improved decision-making. | N/R |
| Barth & Klosinski (2007), Germany | Inpatient child and adolescent psychiatric unit. | 12-year-old female. | Suspected diagnoses of school phobia, social phobia and trichotillomania. | Individual art therapy sessions were conducted over a 2-year inpatient admission. This involved both structured sessions and unstructured free painting. | Qualitative: Clinical observations based on Levels of Emotional Awareness (LEAS, Lane). Stages ranged from Level 1 (affective activation of the autonomic nervous system, questionably conscious, perceived as bodily sensations) to Level 5 (other person has an inner state different from one's own feelings, which can be deduced through empathy). | Clinician reported that the participant moved between Levels of Emotional Awareness 2-5, and showed improved emotional awareness, established healthier and more stable relationships, completed school and training and started a civil service career. | Participant showed great willingness to engage in art therapy and completed independent artmaking alongside art therapy sessions. |
| Bennink et al. (2003), USA | Juvenile justice locked facility for adolescent boys court-ordered to complete a residential program of approximately 4-6 months. | 2 female participants aged 17 and 18 years. | Adolescent-onset conduct disorder (DSM-IV-TR) | Program activities included individual and group-based art therapy, a family empowerment group, academic program, and a vocational program. Art therapy focused on anger management, self-esteem work, and substance abuse education, as well as medication monitoring, coping, social and communication skills. Clients were given structured, directive activities and used drawing, collage, found objects, oil pastels, paints and sculpture. | Qualitative: Clinical observations | Clinician observed improvements in emotional expression, self-esteem, and communication following individual art therapy.  Clinician reported that group participants showed minimal improvements in social, communication, decision-making skills, and self-esteem. This was attributed to therapy-interfering behaviours, time constraints and continuously changing group membership. | Some therapy interfering behaviours noted in group art therapy sessions. |
| Bourry & Barbe (2012), Switzerland | Inpatient adolescent psychiatric unit | 2 participants aged 16 and 17 years. 1 male, 1 female. | Suicide attempt. | Participants had access to the art therapy workshop space two afternoons per week. Land Art occurs in the Unity Garden and involved earth (clay) modelling/sculpture and land art using materials available in nature. | Qualitative: Clinical and phenomenological observations to understanding subjective experience and gain insight into lived experience. | The use of modelling and Land Art helped adolescents confront themes of ephemerality and disappearance, both of their artwork and themselves, symbolising personal struggles. The process translates their internal experiences into physical transformations in their creations, reflecting personal growth and change. | Participants came freely, unlike other therapeutic groups, to discover and work on the mediums made available to them by the art therapist. |
| Briks (2007), Canada | Adolescent outpatient psychiatric unit. | 17-year-old female. | Depression, PTSD, borderline and masochistic personality traits. | Individual therapy sessions were conducted 1-2 times per week, over a 4-year period. Intervention focused on identifying, expressing and releasing emotions. | Qualitative: Clinical observations | Clinician perceived that art therapy provided the adolescent with a safe forum for personal expression and enhanced communication, and observed emotional release, processing and integration of childhood trauma. The participant also maintained regular part-time employment while continuing her academic studies and improving peer socialization. There were no further episodes of self-harming behaviour. | N/R |
| Buchan (2009), Canada | Inpatient children's mental health unit | 9-year-old male. | PTSD, pervasive developmental disorder-not otherwise specified, severe learning disorder, anxiety, phobias, poor impulse control, and poor social skills. | Individual art therapy sessions. Intervention used a non-directive approach alongside play to facilitate self-expression, in addition to developing a bullying intervention plan. | Qualitative: Clinical observations | Clinician reported that art therapy enabled the child to better explore feelings of fear and anxiety and attachment issues. Clinician also observed improved social functioning, a greater sense of accomplishment and functional improvement e.g., attending a swimming field trip. | N/R |
| Burt (1993), Canada | Residential treatment program for displaced youths with multiple failed placements in foster and adoptive homes. | 14-year-old female. | Borderline personality disorder characterised by aggressive, suicidal and deliberate self-harm behaviours. | Individual art therapy sessions. Intervention focused on visual communication and expression using drawing, clay, painting, aiming to support reconnection to Canadian Indian culture. | Qualitative: Clinical observations | Young person’s art featured themes of perceived loss of voice reflecting her experience of cultural displacement and abuse, helplessness and powerlessness, and self-harm functioning as a form of distress tolerance and re-enactment of past trauma. Verbal communication and self-expression improved over time e.g., young person joined an improv theatre group and writing poetry. | Engagement improved over time. |
| Calestreme et al. (2016), France | Therapeutic, educational and pedagogical institute (ITEP) for children with learning and psychosocial disabilities. | 2 participants aged 12-13 years.1 male, 1 female. | Primary diagnosis of psychosis, with varied comorbid diagnoses including bulimia, obsessive compulsive disorder, and global developmental delay. | Weekly 45-minute art therapy sessions were conducted over a 4-month period. Intervention focused on expressive and reflective activities based on a comic strip. | Qualitative: Observation Grid to identify mental activity and clinical evaluation of pictorial mediation in childhood psychosis, and 16-item semi-structured interview based on the Survey of Workshop Activities derived from the Clinical Scale of Mediated Therapies (ECTM2-Q) conducted post-intervention. | Following 7 art therapy sessions, the clinician observed an improved in psycho-affective development and spatio-temporal continuity. Semi-structured interviews showed improved self-awareness, self-reflection and self-expression, reduction in anxiety and inhibition, greater thought fluency and organisation, and greater spatial and motor orientation. | N/R |
| Campbell (1990), N/R | Psychiatric hospitalization in residential treatment setting. | 16-year-old male. | Suicidal and homicidal ideation, and auditory hallucinations. | Individual 60-minute art therapy sessions, twice weekly over a 3-month period. This involved use of clay and creative play with aim of facilitating processing of emotions, normalization of healthy aggression and reduction of defence mechanisms, consistent with a psychodynamic approach. | Qualitative: Clinical observations | Outcomes were unclear. Adolescent explored sado-masochistic themes of power e.g., aggression, victimization and rescue/protection, and sought resolution of early trauma. | Participant was friendly and cooperative in art sessions and expressed enthusiasm for working with art materials. |
| Chong (2015), UK | N/R | 4-year-old female. | Early relational trauma following exposure to domestic violence, foster placements, and maternal depression. | Individual art therapy was conducted over a 5-month period. Involved use of varied materials including paper and clay, focused on holding and containment of client affect. | Qualitative: Clinical observations | Clinician observed that the child showed improved emotional awareness and emotion regulation. Teacher reported a reduction in behavioural disruption and improved confidence, attention span, cooperation and the formation of positive relationships at school. | N/R |
| Clements (1996), UK | Inpatient children's hospital, residential care setting and private practice. | 4 participants aged 6-14 years. 2 male, 2 female. | Referred for mental health problems related to emotional, physical and sexual abuse. | Individual art therapy. Intervention used a non-directive approach to facilitate trauma processing and build insight. Provided choice and control over the image, content, colours, materials, size and aesthetics. | Qualitative: Clinical observations | Clinician reported that art therapy assisted the child in verbally disclosing traumatic abuse and processing negative self-beliefs and low self-esteem, through negotiation of boundaries, sense of control and choice, and emotional expression. | N/R |
| Coskunlu et al. (2018), Ireland | Outpatient child and adolescent community mental health setting. | 2 participants aged 11 and 15 years. 1 male, 1 female. | Participant 1 presented with a depressive episode, generalised anxiety disorder, and insecure attachment. Participant 2 presented with depression with suicidal thinking. | Individual art therapy sessions followed the Vasarhelyi method of child art psychotherapy. Children also received other interventions including cognitive behaviour therapy and antidepressant medications (selective serotonin reuptake inhibitors). | Mixed methods: Clinical observations and self-report questionnaire (Child Depression Inventory) administered to measure depressive symptomatology. | Clinician reported that: - Participant 1 showed an improvement in mood, sleep, self-confidence, self-care, and school functioning, and showed reduced anxiety. Some oppositional behaviour persisted.  - Participant 2 showed improved self-awareness, family communication. There were no further suicide attempts or deliberate self-harm following 9-months of intervention.  Depressive symptoms reduced from the ‘clinical’ to ‘normal’ range on the Child Depression Inventory for Participant 2. | N/R |
| Cunningham & Page (2001), USA | Restricted residential inpatient facility. | 13-year-old male. | Physical aggression, suicidal ideation, and antisocial behaviour. | Individual art therapy once to twice weekly, over a 3–4-month period. Involved traditional talk therapy focused on self-reflection and self-expression around current relationships and behaviour, use of goal-directed board games, and 11 sessions of non-directive attachment-informed art therapy with magazines, pencils and paints. This was in addition to daily group therapy and psychotropic medication | Qualitative: Clinical observations | Clinician reported that the young person expressed a need for safety and security through emotional and behavioural expression and showed improved self-worth. Rapport with therapist improved as evidenced through increasing eye contact and proximity. | Participant overcame initial reluctance and showed improved engagement, trust and self-regulation as sessions progressed. |
| Diamond-Raab & Orrell-Valente (2002), USA | Inpatient child and adolescent psychiatric unit. | 2 female participants aged 12 and 16 years. | Participant 1 was diagnosed with anorexia nervosa and major depressive disorder. Participant 2 had bulimia nervosa, depression, and a history of deliberate self-harm. | Participants completed expressive group therapy 4 times per week integrating art therapy, psychodrama, and verbal therapy. This was in addition to individual art therapy 3 times per week. | Qualitative: Clinical observations | Participant 1 showed a considerable improvement in food intake, no need for nutritional replacements, achieved 95% ideal body weight, and showed improved emotional awareness, expression, and assertiveness. In family therapy, the young person expressed her feelings and needs, and her parents recognised that their interpersonal hostility was manifesting in triangulation of their child. Peer socialisation and maintenance of relationships also improved.  Group and art therapy for Participant 2 supported access to deeply repressed feelings and facilitated improved emotional interpretation and expression, and adaptive coping. She reported a reduction in self-loathing and expressed reduced suicidal ideation and a desire to live. | Participant 1 was initially resistant to nutritional rehabilitation and traditional psychosocial interventions, including individual and family therapy. She was also isolative and refused to interact with peers. For both participants, art therapy, psychodrama, and journaling proved the most effective treatment modalities. Participant 2 also continued art therapy in an outpatient setting. |
| Edan & Knecht-Favrod (2011), Switzerland | Inpatient adolescent psychiatric unit. | 2 female participants aged 15 and 16 years. | Participant 1 presented with a suicide attempt in the context of a trauma history. Participant 2 presented with a suicide attempt, auditory and visual hallucinations, deliberate self-harm, and a severe depressive episode with psychotic symptoms. | Intervention involved a combination of individual and group-based art therapy, and workshops, focused on providing containment and security, consistent with a psychodynamic approach. Group participation was compulsory. Individual art therapy sessions were proposed on request after an evaluation of suitability. | Qualitative: Clinical observations | Clinician reported that: - Participant 1 showed a restored capacity for symbolization, access to more free associations, and facilitated exploration of internal states. - Participant 2 showed an improved capacity for verbalisation of her feelings and needs to her family members, and reduced engagement in maladaptive coping strategies (deliberate self-harm). | N/R |
| Fliegel (2000), USA | Acute adolescent residential and partial hospitalization program (McLean Hospital). | 6 participants aged 13-16 years. 1 male, 6 female. | Various presentations including anger management, poor social skills, depression, deliberate self-harm, suicidal ideation, substance abuse, sexualised behaviour, court involvement, learning disabilities, PTSD, anxiety, eating disorders, psychotic thought processes, and/or poor life management skills. Some adolescents had a long history of family problems, including child abuse, addiction, mental illness, and limited parental capacity. | Individual and group art therapy were conducted, in addition to exposure to a wide range of community-based activities (e.g., creative writing workshops, concerts, art-making workshops, and trips to public libraries and museums). Intervention was informed by a psychodynamic approach to support identity formation and a sense of self. | Qualitative: Clinical observations | Clinician reported that: - Participant 1 showed a reduction in deliberate self-harm, improved self-esteem, and enrolled in an art program. - Participant 2 showed reduced oppositionality and an improvement in prosocial behaviour.  Participant 3 was linked in and hopeful about attending twice weekly dance classes. Participant 4 showed a reduction in suicidality, and improved sense of purpose, and enrolled in a film class.  Group participants attended a music concert and showed good crowd participation, reduced anxiety and panic. | N/R |
| Gander et al. (2018), Austria | Inpatient psychiatric unit. | 16-year-old female. | Anorexia nervosa in the context of deliberate self-harm, obsessive compulsive traits, depressive episodes and depressive personality disorder, and borderline personality disorder traits. | Individual art therapy sessions. Intervention techniques included the triptych (three-part picture), free expressive painting, body-cantered work using body painting, collages and adhesive pictures, the progressive therapeutic mirror image, the body image and the clay design. | Qualitative: Clinical observations | Attachment issues and attachment-related traumas were reflected in the artwork of the patient with anorexia, facilitating recovery from these potentially traumatizing events. | N/R |
| George & Kasinathan (2015), Australia | Secure forensic psychiatric hospital providing acute, subacute and rehabilitation care to young civil, correctional and forensic patients. | 11 male participants aged 15-20 years. | Primary diagnosis of schizophrenia or schizophreniform disorder (n=9) and bipolar affective disorder (n=1), with comorbid conduct disorder (n=10) based on DSM-IV. One young person had no mental illness and was admitted for diagnostic clarification. Sentence length, legal status and the clinical need for psychiatric admission varied between participants. | Weekly group art therapy sessions over a 10-weeks period. Participants were provided therapeutic space, tools and techniques to create a design that reflected their selves, their unit and complemented the courtyard in a graffiti-style work. | Qualitative: Anecdotal feedback. | Outcomes were unclear. | Mural art therapy was generally deemed acceptable. Two adolescents prematurely and voluntarily terminated participation. Feedback from staff members and adolescent patients admitted following the completion of the mural was uniformly positive. |
| Gerteisen (2008), Alaska, USA | Residential treatment centre. | 11-year-old male. | Foetal alcohol spectrum disorder and childhood trauma. | Weekly individual and group art therapy sessions over a 9-week period. Young people were provided with a sketchbook, scented watercolour markers, pencils, and collage materials and engaged in structured art tasks. Intervention focused on improving self-esteem, social relationships, interpersonal skills, emotional expression, body awareness and self-esteem. | Qualitative: Clinical observations | Clinician reported that drawing supported the child in overcoming feelings of inadequacy associated with FASD-related symptoms while also addressing trauma through telling his story of fear, worry, hurt, anger, inadequacy, and overall victimization. | N/R |
| Gmitrowicz et al. (2012), Czechoslovakia | Inpatient adolescent psychiatric unit. | 18-year-old female. | Initially diagnosed with depressive episode in context of bipolar affective disorder (ICD-10), suicidal thoughts and tendencies. Diagnosis changed to schizoaffective disorder with support from art therapist observations. | Individual art therapy sessions. This focused on diagnosis, communication, integration and catharsis, consistent with a psychodynamic approach. | Qualitative: Clinical observations | Clinician observed that the participant showed greater emotional expression and regulation, social functioning, self-awareness, and improved interaction with staff. | Participant willingly communicated only with the art therapist, discussing her works, also painted on the walls and windows of the hospital ward. Patient selected only individual art therapy sessions and refused group participation. |
| Hamernik (2002), USA | Inpatient child and adolescent psychiatric unit. | 3 participants aged 12-18 years. 1 male, 2 female. | Varied diagnoses including depression, suicidal ideation and attempts, drug use, ADHD, conduct disorder and eating disorders. | Adolescents participated in group art and play therapy. Intervention techniques involved three activities (1) 'colour your life' technique, choosing colours to represent feelings and mapping to timeline, (2) touch and smell game, (3) chicken soup story group. Family art therapy sessions were also offered. | Qualitative: Clinical observations | Clinician reported that children demonstrated improved insight, motivation for change, as well as improved capacity for verbal and emotional expression. | N/R |
| Hanes (1997), USA | Inpatient psychiatric unit. | 6-year-old female. | Aggressive behaviour, uncontrollable tantrums, and behavioural problems including sexually inappropriate behaviour. | Twice weekly compulsory group art therapy sessions over a 6-week period. Group size varied from 3 to 5 participants. Intervention involved an orientation to the art studio, a non-directive approach and free choice of materials. | Qualitative: Clinical observations | According to the clinician, artmaking facilitated the child’s exploration of unresolved, confusing and chaotic emotions, and communication around feelings of mess. On termination of sessions, the child relinquished artwork possibly representing relinquishment of painful emotions and life experiences. | The participant often entered sessions with enthusiasm and excitement. |
| Hanvey & Tepper-Lewis (2019), USA | Child and adolescent unit of a comprehensive psychiatric emergency centre. | Children and adolescents aged 5-17 years. | Various diagnoses necessitating up to 72 hours observation. | Single standalone group art therapy session. The program took a psychodynamic dual-modality approach to providing structured sessions to facilitate symptom relief, improve interpersonal skills and self-efficacy. Sessions commenced with intention-setting, verbal discussion, before progressing to a dance/movement exercise with a dance/movement therapist, then the ar therapist facilitate exercises using imagery and symbolic play, and use of mural, markers, crayons and oil pastels to enable making of one unified artwork, followed by verbal processing of the work. | Qualitative: Clinical observations | Facilitators observed group bonding, cohesiveness and social interaction through movement or visual art. Participants showed improved assertiveness and a more positive attitude towards their inpatient hospital stay, greater prosocial behaviours, and a greater sense of belonging. | Participants perceived art psychotherapy as a safe and structured environment. Group members were willing and enthusiastic, free in spontaneous movement and self-expression. However, some group members vocalized feeling anxious and resistant to engaging in exercises due to disinterest or fear of judgement. |
| Harnden et al. (2004), Canada | Outpatient mental health setting | 14-year-old female. | Suicidal ideation in context of comorbid depression and PTSD. | Weekly 60-minute individual art therapy over a 48-week period. Intervention focused on emotional containment and expression, improving self-esteem and identity formation, and took place alongside a multidisciplinary team intervention. | Qualitative: Clinical observations | Clinician reported that art therapy resulted in increased self-esteem, decreased hopelessness, and suicidal ideation, reduced post-traumatic stress symptoms and improved school and social functioning. | N/R |
| Henley (2007), USA | N/R | 16 children aged 9-16 years. | Paediatric bipolar disorder and comorbidities including ADHD and autism spectrum disorder. | Individual art therapy sessions over a 2 ½ year period, ranging in frequency from weekly to monthly depending on need and engagement. Intervention involved non-directive and spontaneous use of art materials, and pre- and post-artmaking reflection. | Qualitative: Clinical observations | Clinician reported benefits for emotion regulation, cognitive organisation, and ego strengthening, but more variable changes in self-awareness. | N/R |
| Hetherington & Gentile (2022), Italy | Private practice studio | 13-year-old male. | PTSD | Weekly 60-minute individual sensorimotor art therapy sessions over a 2-year period. This integrated somatic experiencing (a technique for treating trauma) within a psychodynamically oriented intervention. Intervention also involved bi-weekly parent sessions focused on managing behavioural difficulties related to post-traumatic stress, and ongoing psychology input and group support meetings. | Qualitative: Clinical observations | Participant reported the two most helpful aspects of therapy to include playing football and receiving a gift from the therapist. Clinician viewed benefits as improved emotion regulation, bodily awareness, sense of safety, emotional expression and negotiation of interpersonal and intrapersonal boundaries, autonomy and reduced post-traumatic stress. | Participant initially described art therapy sessions as useless, however following one year showed greater openness and emotionality. |
| Horovitz (1981), USA | Therapeutic nursery of a child and family community mental health centre. | 5-year-old male. | Infantile autism, residual type (DSM-III) in context of attachment and developmental trauma. | Weekly individual art therapy sessions over a 16-month period. Intervention involved use of drawing, painting, clay to support healthy ego maturation, consistent with a psychodynamic approach. | Qualitative: Clinical observations | Clinician observed improvement in communicative abilities, emotional expression, cognitive and academic skills, and self-esteem. Clinician purported that art therapy and symbolic play allowed processing of early infantile trauma. | N/R |
| Horovitz (1983), USA | Therapeutic nursery of a child and family community mental health centre. | 3 participants aged 4-5 years. 2 male, 1 female. | Participant 1 was diagnosed with oppositional reaction of childhood, later changed to anxiety reaction of childhood. Participant 2 was diagnosed with unsocialized reaction of childhood later changed to borderline psychosis. Participant 3 was diagnosed with overanxious reaction of childhood (DSM-II and DSM-III). | Weekly art therapy sessions for 6 months to 1.5 years, ranging from 29-59 sessions total. Involved drawing and use of clay materials. Additionally, children were assigned an educational therapist, sociotherapist, family caseworker, psychologist, and psychiatrist. | Qualitative: Clinical observations | Outcomes were mixed. Clinicians observed that all children showed gains in cognitive, emotional and verbal development. Two children showed an improvement in socialisation and academic skills, whereas one child experienced ongoing difficulties in these areas. | Participant resistance to art therapy reduced over time as engagement improved. |
| Howie et al. (2002), USA | Comprehensive family support program for children and families affected by the 9/11 World Trade Center tragedy. | 10 participants aged 3-13 years. 4 male, 6 female. | Acute traumatic stress, at risk for PTSD (DSM-IV-TR). | Group art therapy for children and their families. Intervention aimed to assist in witnessing and processing trauma and creating safety. This involved use of various materials including paper, pencils, markers, pastels and modelling clay with an open-ended unstructured approach. Additionally, families were offered grief and family counselling, day care services, assistance with wills, funeral arrangements, and psychological evaluation and treatment. | Qualitative: Clinical observations | Clinicians reported that many children benefitted from opportunity to process fears, and that art therapy supported in creating safety and connection with other trauma victims. Some children found solace in creative and metaphoric solutions to their grief and loss, while others had more difficulty processing and resolving their loss. | Each child interacted with and used the art materials uniquely. |
| Kelemen & Shamri-Zeevi (2022), USA | Therapeutic day school for students recovering from mental health conditions. | 13 participants aged 13-17 years. 4 male, 9 female. | Referred for academic decline, psychiatric issues impairing success in mainstream school setting, or withdrawal and refusal to leave their bedroom for extended time periods. Varied presentations including anxiety, depression, low self-esteem, and attachment trauma and school truancy. | Weekly group art therapy sessions over a 10-month period lasting 1.5-3 hours in an open studio. Intervention used a non-directive humanistic approach to improve self-exploration, peer interaction and transition back to community. Intervention used various art materials including lead and coloured pencils, markers, white glue, oil pastels, gouache and paint brushes, clay and plasticine, scissors and magazines for collage, paper, image-making, reparative side-by-side play, and/or mutual aid. Art intervention occurred alongside student activities like meal preparation, dining, kitchen clean up, animal-assisted therapy, individualized education, field trips, and community service. | Qualitative: Clinical observations. | Clinician reported that: - Participant 1 showed an improvement in male peer relations, self-esteem and identity development, and a reduction in social anxiety.  - Participant 2 showed increasing safety and trust in interaction with adults, and reduced anxiety. | N/R |
| Kim & Ki (2014), South Korea | Outpatient mental health treatment setting. | Female third-year high-school student. | Presented with somatisation symptoms and neurasthenia. | 21 individual art therapy sessions 3-4 times a week lasting 40-90 minutes from July-August 2012. Involved drawing, free use of materials, body movement, stretching and walking meditation components. Sessions focused on investigating the subject's mental state and family situation and improving emotional expression and recovery of bodily sensations. Sessions took place at an art therapy lab, school sports field and park. | Mixed methods: Self-report measure of somatisation (20-item Alexithymia Scale Questionnaire and Symptom Checklist-90 Revision), and Body Image Projective Drawing Test given pre-test, post-test and 4-month follow-up. Content analysis of each session was also undertaken. | Reduction in alexithymia on Alexythymia Scale Questionnaire (pre-test score=73, post-test=45, follow-up=32).  Reduction in somatisation symptoms on Symptom Checklist-90 Revision (pre-test score=43, post-test=23, follow-up=25). Results of the Body Image Projective Drawing Test showed improved emotional expression and reduced somatisation via colour distribution and choice. | N/R |
| Kozlowska & Hanney (1999), Australia | Tertiary child psychiatry treatment setting for children aged 2-8 years. | 2 participants aged 5 and 6 years. 1 male, 1 female. | Oppositional behaviour involving physical and verbal aggression. Families referred to the program have usually failed to benefit from interventions at the local community health centre, with their paediatrician or other health professionals. | Participants completed 1-3 x 60-minute weekly assessment sessions with the whole family present. Following this, families were tasked with completing a family-cantered interactive art exercise using multiple materials (pastels, glue, clay, magazine cuttings), as informed by attachment theory and family therapy. The therapist observed from behind one-way mirror, then facilitated family discussion and identified goals and homework. Art intervention occurred within a systemic treatment framework that involved family therapy, family admission, specific pharmacotherapeutic or other psychological approaches and involvement of some children in a day program. | Qualitative: Clinical observations of structural family therapy data (parenting skills, roles, hierarchies, boundaries, alliances and coalitions), family interactions and individual style of interaction, and child's developmental level. | Clinicians observed that art therapy highlighted problematic patterns of family interaction (e.g., shared parenting role between child and parent, patterns of over-responsibility, compliance to reduce demands on family, parenting conflict and inconsistency, and sibling rivalry). Art therapy also supported identification of collaborative therapeutic goals, whilst modelling limit-setting strategies. | Overall positive experience. Only a small number of families (8/100) were unable to use the art exercise. |
| Kozlowska & Hanney (2001), Australia | Outpatient setting for children aged 4–8 years with psychiatric disorder. | 5 participants aged 5-8 years. 4 male, 1 female. | Primary diagnosis of PTSD (DSM-IV, ICD-10) related to exposure to cumulative traumatic experiences involving threats to primary attachment figures in the context of conflictual, violent or unresolved parental separation. Comorbid diagnoses included depression (n=5), oppositional defiant disorder (n=4), learning difficulties (n=3), and conduct disorder (n=1). | Weekly 60-minute group art therapy sessions over 7-weeks. Therapy took an attachment-informed approach and utilised aspects of cognitive-behavioural therapy. Sessions aimed to facilitate desensitization and processing of traumatic memories. All participants were experiencing concurrent family-based treatment and had previous treatments including outpatient family therapy, medication, admission to a therapeutic day programme, inpatient family work and home visits by nurses, aiming to ensure safety and stability within the family context. | Qualitative: Clinical observations of participant engagement and symptom profiles. | Art therapy group facilitated exposure to traumatic cues in a less direct manner, facilitating desensitization of anxiety and unpleasant body sensations, processing of parental separation, and labelling and articulation of emotional states using art and language in a safe and supportive communal environment. At the end of multimodal treatment, all children successfully returned to school and only one family required involvement of children protection services. | Young people enjoyed coming to the group and using the art materials. When the group ended, young people expressed disappointment and frequently asked if another group could be run. |
| Lindinger & Karwautz (2015), Vienna | Inpatient child and adolescent psychiatric unit | 5 adolescent females aged 15 years. | Varied diagnoses including bulimia nervosa (n=2), anorexia nervosa restrictive subtype (n=1), and anorexia nervosa binge/purging subtype (n=1). | Individual art therapy sessions conducted twice weekly. | Qualitative: Clinical observations | Clinician reported that patients showed strengthened sense of self and self-confidence, increased willingness to talk about their experiences, and a decrease in disordered eating compulsions. | Art therapy supported young people in following creative desires, at first hardly noticing the therapeutic process and overcoming uncooperativeness or defensive attitudes. |
| Mallay (2002), Canada | Community outreach program involving home-based therapy. | 10-year-old male. | PTSD and acquired brain injury diagnosed following motor vehicle accident. | 15 individual art therapy sessions over a 6-month period. Frequency varied from weekly to fortnightly. Metaphor, graphing and drawing systems information were used to facilitate self-expression (e.g., body mapping, feeling chart, journalling), and exploration of family system dynamics. Intervention also aimed to assist in managing numbing and avoidance behaviour and allow desensitization and cognitive restructuring if necessary. | Qualitative: Clinical observations | Clinician reported improvements in affect regulation, sleep, engagement in extracurricular activities and social functioning, improved control over hallucinatory experiences and a reduction in psychophysiological symptomatology. | N/R |
| Mazloomian & Moon (2007), USA | Residential treatment centre for male adolescents who have committed a sexual offense. | 2 adolescent males. | Varied mental health diagnoses in the context of a childhood sexual abuse history. | Individual art therapy. Intervention involved deliberate welcoming, gathering materials, preparing the space, making art, cleaning up, and wrap-up procedures. This provided an action-oriented environment and aimed to support adolescent males in finding male mentoring figures, and internalising positive messages regarding self-worth. Additionally, participants could access psychology, social work and counselling, and completed cognitive behaviour therapy, recreational and music therapy. | Qualitative: Clinical observations | Participant 1 showed improved emotional expression and mental health following 4-years of art therapy.  Participant 2 showed improved self-esteem, positive self-image and interpersonal trust following 20 weekly of art therapy sessions. | Participants engaged willingly in artmaking. |
| McGann (1999), USA | Outpatient adolescent day treatment program. | 15-year-old female. | Presented with depression, homicidal ideation, poor interpersonal skills, poor school performance, anxiety, and language difficulties in the context of developmental trauma. | Weekly individual art therapy sessions over an 18 month-period. Focused on facilitating expression of homicidal rage and aggression. This was provided in addition to weekly talk-based therapy, monthly family therapy, and psychotropic medication. | Qualitative: Clinical observations and parent and child self-report. | Participant made significant gains in accepting and developing trusting relationships and building a sense of safety interpersonally and intrapersonally, through increased distress tolerance. Participant showed a reduction in homicidal ideation and decreased levels of destructive, aggressive behaviour at home. Family relationships remained strained. | Abrupt termination of treatment due to limited parental capacity. |
| McMurray & Schwartz-Mirman (1998), Israel | Therapeutic boarding school for children at-risk. | 9-year-old female. | Developmental trauma. | Individual art therapy and symbolic play over a 3-year period using a psychodynamic approach to interpretation of artwork and transference interactions within therapeutic relationship, and healthy ego development. | Qualitative: Clinical observations | Facilitated verbal disclosure of abuse, reduction in oppositional and defiant behaviours and increase in cooperativeness. Artwork moved from compulsive fixed pattern of dirtying and cleaning materials to figurative organized painting. | N/R |
| Milia (1996), USA | Outpatient day treatment program. | 15-year-old female. | Presented with depression, deliberate self-harm and suicide attempts. | Individual art therapy sessions. Involved use of drawing, clay sculpture, and pastels. Focus was to facilitate communication around self-harm and provide opportunities to exercise destructive and integrative urges consistent with psychodynamic approach, as an adjunct to talk-based therapy. | Qualitative: Clinical observations | Clinician reported improvements in emotional expression, processing and integration of traumatic memories, and individuation. | N/R |
| Miller (2007), USA | Outpatient day treatment program for adolescent girls aged 12-20 with severe emotional and behavioural problems. | 13-year-old female. | Presented with developmental trauma disorder, including severe emotional and behavioural difficulties and learning disorders. | Weekly 45-minute individual art therapy sessions over a 4-month period. Participant attended 12 out of 15 sessions. Sessions were non-directive and client centred. Additionally, participants had a full-day therapeutic milieu including individual and group verbal therapy, art therapy, and family counselling, and school. | Qualitative: Clinical observations | Art therapy sessions supported the adolescent in building a sense of trust and safety, as well as expressing primary emotions and processing traumatic re-enactments. Adolescent also showed increased feelings of mastery, competence, and ego strength, generalising to improved self-esteem and peer relations. | N/R |
| Moon (1999), USA | Inpatient child and adolescent psychiatric unit. | 3 adolescents. | Varied mental health diagnoses. | Individual art therapy utilising responsive artmaking. | Qualitative: Clinical observations | Outcomes were unclear. | Art therapy assisted in deepening therapeutic rapport, increasing treatment engagement, and circumventing defence mechanisms. |
| Naumburg (1945), USA | Inpatient child and adolescent psychiatric unit. | 9-year-old male. | Primary behaviour disorder (neurotic type). | Individual art therapy sessions weekly for approximately 6 months. Intervention techniques involved drawing, painting and sculpture, in addition to talk therapy and sexual education. | Qualitative: Clinical observations | Participant expressed anger and intolerance towards primary attachment figure's failures through death scenes. Over time, participant showed a diminished preference for war scenes, indicating a symbolic release of long repressed conflicts, hostility, insecurity and anxiety. Participant also processed traumatic experiences through completion of picture series. | Participant remained tense and hyperkinetic yet was eager and interested in the artwork. |
| Naumburg (1946), USA | Inpatient child and adolescent psychiatric unit. | 10-year-old male. | Primary behaviour disorder (neurotic type). | Individual 30–60-minute art therapy sessions once to twice weekly for 6 months. Intervention techniques involved drawing, painting and sculpture, in addition to talk therapy and sexual education. | Qualitative: Clinical observations | Participant showed increasing maturity by moving from copying drawings to drawing own images of animals. Participant showed an increased ability to express himself spontaneously in creative art forms and to improvise freely in both verbal and pictorial fantasies, assisting resolution of conflictual home environment and age-appropriate sexual conflicts. | Participant's initial attitude of doubt and suspicion gradually developed into confidence and friendliness towards the interventionist. |
| Nielsen et al. (2021), Australia | Inpatient residential adolescent psychiatric unit. | 3 adolescents. | Severe depression, suicidal ideation, and self-harm (Participant 1). Chronic depression, suicidal ideation, and self-harm within the context of substance use and school refusal (Participant 2). Depression, PTSD, and possible psychosis (Participant 3). | Participants completed 3 x 60-minute weekly assessment sessions. If the family proceeded, family art therapy sessions were offered on an unlimited basis. Intervention involved the parent and young person working separately on artworks, with time for discussion. This focused on symptom reduction, improving parent-child attunement, and parental self-care. Additionally, adolescents complete a daily therapy program, individual, family talk-based therapy, and optional music therapy. | Qualitative: Clinical observations | Clinician reported: - For Participant 1, art therapy supported identification of parent-child attachment issues (e.g., mutual caretaking) and parental mental health issues. Parent modelled more adaptive coping strategies. Adolescent became more independent, peer-oriented, engaged in ward program, and had reduced suicidal ideation and deliberate self-harm. - For Participant 2, art therapy allowed parent to identify own mental health difficulties. Parent-child relationship and communication improved. Young person showed improved mental health, school attendance, peer socialisation, and healthy independence.  - For Participant 3, art therapy supported parents in acknowledging and understanding attachment problems, their child's distress and maladaptive coping strategies. Young person expressed distress safely and showed increased trust in parents ability to manage low mood and auditory hallucinations, as well as successful school transition on discharge. Parents increased emotional attunement to child, awareness of own intergenerational trauma, and sought own mental health treatment. | One parent disengaged from family-based art therapy. In another family, both the parent and child expressed interest in pursuing ongoing art therapy and reported finding art therapy enormously beneficial. Another family reporting having a positive experience of art therapy. |
| Nikolova et al. (2016), France | Inpatient child and adolescent psychiatric unit. | 6 adolescent females. | Anorexia nervosa restrictive type (n=3), psychotic decompensation (n=2) and neurotic disorder of the hysterical type (n=1). | Group art therapy workshops. Sessions began with relaxation and warm-up and body awareness, progressing to drawing, painting or modelling. | Qualitative: Clinical observations | Participant reported improved self-expression and a sense of freedom, beauty and inspiration. | N/R |
| Peake (1987), N/R | Outpatient treatment setting. | 8-year-old male. | Generalized anxiety disorder with depressive and obsessive features, and enuresis. | Individual 60-minute art therapy sessions used a nondirective and unstructured approach and took place over an 18-month period. | Qualitative: Clinical observations | Participant showed reduced aggression (identification with the aggressor combined with the masochistic-victim role) and reduced splitting, allowing greater integration of his identity. | N/R |
| Potash (2009), Hong Kong | Private practice studio. | 2 participants aged 13 and 15 years. 1 male, 1 female. | Self-harming behaviour, anger management, and strained family relationships (Participant 1). Oppositional behaviour, anger management issues, and difficulties with verbal processing (Participant 2). | One-hour weekly art therapy sessions for approximately 2-months. Artmaking involved unstructured nondirective drawing to facilitate creative self-expression. Accompanied by parenting skills and behaviour management strategies. | Qualitative: Clinical observations | Clinician reported that : - Participant 1 showed improved self-concept and reduced perfectionism. Deliberate self-harm behaviours continued to fluctuate.  - Participant 2 showed a reduction in oppositional behaviours and increase in mood, however noted ongoing use of internet pornography and verbally aggressive social media use. | Participants were responsive and observed to enjoy art therapy. |
| Powers & Langworthy (1978), USA | Inpatient adolescent psychiatric unit. | 7 participants aged 13-17 years. 4 male, 3 female. | Various diagnoses including borderline personality disorder, conduct disorder, schizoid personality disorder, acute psychosis with paranoid ideation, anorexia nervosa, and suicidality. | Short-term group and individual art therapy of 3-6 months aimed to promote awareness, insight and expression of emotional states and interpersonal awareness. This formed part of a comprehensive psychodynamic treatment program using a social psychiatric therapeutic community, individual and family therapy, group therapy, occupational therapy, and recreational therapy. | Qualitative: Clinical observations | Group-based art therapy facilitated emotional expression in the first project, but not the second project. Individual art therapy supported a participant in expressing feelings regarding her body-image, identity, dependency and institutionalisation. | Cohesive, cooperative and warm interactions were observed amongst group members in the first project. Participation was minimal in the second project. In individual therapy, the participant reported her artwork was one of the most helpful aspects of treatment. |
| Raghuraman (2000), USA | Inpatient psychiatric unit. | 14-year-old male. | Presented with deliberate self-harm, violent outbursts, oppositional behaviour, and non-compliance with Type 1 diabetes management. | Weekly individual and twice weekly group sessions for 60-minutes each, over a 6-month period. Intervention used various creative materials and aimed to provide an outlet for anger and depression, improve adaptation to diabetes and hospitalization, increase self-esteem, help accurately assess impacts of diabetes on body-image and identity formation, and provide a sense of mastery and control. This occurred alongside the academic and physical exercise ward program, paediatrician review, social work therapy, nursing and psychology. | Qualitative: Clinical observations | Clinician reported that in 6-months, the participant made progress only during the first month. Participant gained a sense of mastery and control within the limited and restricted ward environment through problem-solving skills and decision-making during sessions. Participant also showed increased self-awareness and self-esteem through emotional expression and positive self-statements. | Participant lacked enthusiasm and motivation to participate in verbal discussions regarding art therapy. Required frequent prompting to attend sessions or arrived and left at unpredictable times in the final three weeks. |
| Robb (2002), USA | 6-week summer program for Russian children from orphanages to strengthen potential adoption relationships with American and Canadian families and support transition into American culture. | Children and adolescents. | Children endured the extreme effects of institutionalization. Many had medical or mental health impairments (including attachment disorders and PTSD) and experienced traumatic events such as parental suicide or severe abuse. Most children appeared anxious and depressed and had minimal concentration abilities. One child made suicidal gestures. | Small-group and individual art therapy sessions conducted at an open studio over a 6-week period. Incorporated movement and other nonverbal communications, in addition to daily physical activities, visits to American cultural sites. Aimed to promote self-expression, strengthen decision-making, adaptability to change and non-verbal expression of needs. | Qualitative: Clinical observations and the Face Stimulus Assessment task, an art-based projective task completed pre-and post-program. | Only 7 children completed pre and post Face Stimulus Assessments due to limited structure in program design. This could not be analysed. Most children showed limited progress or change in their artwork. Some found a benefit of art therapy in addressing anxiety-provoking issues, such as immersion in American culture and adjusting to the adoption process. | Variable. For some children, the open studio provided the structure needed to feel safe in self-expression. Other children were more tenacious or did not respond to an unstructured approach. One child became more aloof, guarded, edgy and withdrawn over time. |
| Roijen (1991), Denmark | Child psychiatric department. | 11-year-old female. | Presented with psychosomatic fever, anxiety, headache, stomach pains, and reported command hallucinations. | 6-10 sessions using a transcultural family therapeutic approach involving art therapy and individual therapy. The focus of therapy was to support symptom reduction via structural realignment of the parent and child subsystems within the family, and use of drawing and symbols as a communication form independent of language constraints to process family stressors around illness, death and recovery. | Qualitative: Clinical observations | Clinicians observed an improvement in the child and father’s mental health symptoms, and family functioning. | Interventionist reported the therapeutic atmosphere was tense and that the father had difficulty participating in drawing but was active in discussion. |
| Rupa et al. (2014), India | Inpatient hospital setting | 7-year-old male. | Adjustment disorder with mixed disturbance of emotion and conduct. | Individual daily expressive therapy sessions involving play, art and storytelling over a 3-month period. This was followed by weekly and bi-monthly follow-up. Intervention involved non-directive play to support improved autonomy via choice over toys, music, storytelling and art to rebuild future orientation and hope, and group play. This occurred alongside medication management. | Qualitative: Clinical observations | Clinician reported that the child’s aggression reduced significantly, social skills and frustration tolerance improved, as did functionality in activities of daily living. The child developed healthy ways to resolve his grief. In subsequent follow ups, some behaviour problems persisted but improvement was maintained in mood, aggression and anger outbursts at home and school. Two years later, maintenance of therapy gains was complicated by ongoing psychosocial, legal and economic stressors. | N/R |
| Seftel (1987), USA | N/R | 12-year-old male. | Schizoid and autistic-like features. | Individual art therapy using varied materials, primarily clay, consistent with a psychoanalytic and Gestalt approach. | Qualitative: Clinical observations | Child directed destructive feelings into symbolic play, which supported self-expression, mastery and psychic integration. | N/R |
| Sikes & Kuhnley (1984), USA | Inpatient child psychiatric unit. | 5-year-old male. | Disorganised schizophrenia (DSM-III). | Multimodal treatment involved play therapy using structured materials (e.g., doll house) to assist in verbally labelling emotions, organising thoughts, reality testing, and managing internal conflicts. Art therapy took a structured approach focusing on releasing negative affect, facilitating nurturance needs, (e.g., person drawing, kinetic family drawing) and managing abandonment fears. Additionally, behaviour management plans and transition planning were undertaken, alongside weekly multidisciplinary team meetings. | Qualitative: Clinical observations | This coordinated multimodal treatment approach assisted in supporting age-appropriate functioning, promoting emotional expression and conflict resolution, building sense of self, self-esteem, ego boundaries and reality testing, and decreasing symptoms of withdrawal, disorganisation and acting out. Gains were maintained at 18-month follow-up through ongoing family therapy in outpatient treatment. | N/R |
| Small & Greenway (1988), UK | Regional inpatient child psychiatric unit. | 9-year-old male. | Presented with anxiety and severe psychosomatic gastrointestinal symptoms. | 12 60-minute weekly art therapy sessions. Intervention aimed to facilitate exploration of childhood fears and grieving of loss of twin brother, and occurred alongside the regular treatment milieu, parent and family therapy. | Qualitative: Clinical observations | The child showed a reduction in psychosomatic gastrointestinal symptoms, resumption of normal development and social progress (e.g., returning to school, making friends, and improvement in family relationships). Family sessions reduced parental anxiety, facilitated grieving, and appropriate limit-setting. | N/R |
| Speltz (1990), USA | Psychiatric population within mental health setting. | 3 participants aged 15-17 years. 2 male, 1 female. | Participants had varied diagnoses including major depression and borderline personality disorder. All young people participated in satanic practices. | Individual art therapy. Sessions focused on releasing 'satanic' feelings and re-associating anger with real objects or persons to work through in psychotherapy, consistent with a psychodynamic approach. | Qualitative: Clinical observations | The first child gained more self-control but remained interested in satanism. The second child showed a reduction in satanic fantasy, by using fictional characters to express feelings. For the third child, art therapy supported self-expression and provided a coping mechanism to contain aggressive and violent impulses, and preserve self-esteem. | N/R |
| Stanley & Miller (1993), USA | Residential group home. | 15-year-old male. | Presented with behaviour problems including verbal aggressiveness, behavioural outbursts, property destruction, noncompliance and school refusal, in context of self-identity and interpersonal issues. | Weekly 45-minute individual art therapy sessions over an 8-week period. Four sessions involved a prescribed art-based task, and four sessions involved non-directive free drawing using poster board, paints, crayons, pencils and drawing paper. | Mixed methods: Self-report questionnaire (School Form of the Coopersmith Self-Esteem Inventory) administered pre-post intervention in addition to clinical observations, and qualitative interviews with staff. | Adolescent showed improvements in self-esteem (both observationally and via questionnaire), including a more positive self-concept and appraisal of the parent-child relationship. Interviews with staff members suggested decreases in maladaptive behaviours, including a marked reduction in destructiveness, verbal and physical aggression at home and school, and improved school attendance and participation. | Participant looked forward to art therapy and enjoyed participating in sessions. |
| Steinhardt (1995), Israel | Outpatient child therapy setting. | 7-year-old male. | Borderline psychotic. | 1-hour group art therapy and 30-minute individual art therapy sessions over a 3-year period. Intervention involved use of drawing, painting, collage, clay and plasticine use, music, play, puppetry and storytelling focused on providing a safe space for expressing aggressive emotions, exploring and clarifying taboo topics. This occurred alongside family therapy. | Qualitative: Clinical observations | Clinician reported that participant had a reduction in specific phobias (dogs and rain), an improved sense of self, improved emotional expression and regulation, and better peer relations. Family therapy facilitated changes in family structure and strengthening of parental subsystem. | N/R |
| Testa & McCarthy (2004), USA | Inpatient child psychiatric unit. | 3 male participants aged 11-12 years. | Presented with multiple traumas in context of early neglect, parental abandonment, and multiple foster home placements, as well as several hospitalizations for extreme aggression and violence. Two children also had histories of suicidal ideation and behaviour. | Weekly 60–90-minute group art therapy sessions over a 12-week period. Group sessions started with check-in, brief-warm up exercise, then painting and assembling images of a memorial mural about the destruction of the World Trade Center. | Qualitative: Clinical observations | Creation of a mural supported participants in processing and increasing tolerance of traumatic memories and histories. Participants experienced strong group identification, greater self-efficacy, and a healthier attachment to protective adult figures. Participants were able to communicate a powerful message of hope and remembrance for themselves and for viewers while rebuilding trust in their future and world. | Participants enjoyed the mural project. Other patients in the unit asked to become involved in the mural project, and the final product received acclaim as an important contribution to the hospital community. |
| Van Lith (2008), Australia | Transition from residential inpatient unit to psychosocial residential rehabilitation setting. | 16-year-old female. | Provisionally diagnosed with borderline personality disorder in context of family conflict, deliberate self-harm, suicidal ideation and suicide attempts, pseudo hallucinations, low mood, and frequent limited food intake. | 11 individual art therapy sessions twice weekly, and daily visual journalling over a 6-week period. Art therapy took an open-ended, person-centred approach, and involved verbal discussion, written responses, and visual creation of images with free choice of materials. This occurred alongside intensive mental health support and case management. | Qualitative: Phenomenological analysis to identify patterns and themes in participant verbal dialogue, text, artmaking, visual journal and post-session reflections. Data analysis identified themes and repeated visual features and enhanced description of the participant’s experience. | Key themes included: a sense of uncertainty, exploring difficult emotions through art therapy, the emergence of a character to assist the release of strong emotions, deeper discovery of the inner self, and becoming an individual. Visual journal facilitated self-expression and guided self-identity and functioned as a self-therapy tool following end of therapy. | Participant regularly attended sessions and completed her visual journal daily. Young person progressed from emotionally flat, difficult to engage, and resistant in initial sessions, to more relaxed, open to discussion, independent in exploration of topics, and confident in initiating discussion as well as setting boundaries. |
| Vick (1999), USA | Inpatient adolescent psychiatric partial hospitalization program. | 10 participants aged 12-17 years. 3 male, 7 female. | Primary psychiatric diagnosis. A significant percentage of young people also had substance abuse histories. | 45-minute art therapy groups. Six types of prestructured art elements (magazine pictures, magazine words, photocopied images, cut and torn paper, traced shapes, and partial drawings) were used to facilitate self-expression. | Qualitative: Clinical observations | Clinician reported that participants used art therapy for self-expression and exploration of identity and their current developmental phase. | N/R |
| Waller (2006), UK | Inpatient adolescent psychiatric unit. | 2 female adolescents aged 11-15 years. | Presented with deliberate self-harm and psychosomatic symptoms. | Group art therapy sessions during course of hospital admission Involved use of drawing, clay making, and painting to facilitate healing, sublimation of feelings, and prosocial communication, consistent with psychodynamic approach. | Qualitative: Clinical observations | One participant showed improvements in behaviour, trust and self-esteem. The other showed improved peer relationships and school enjoyment. | N/R |
| Wardle (2023), Scotland | Outpatient mental health treatment setting. | 6-year-old male. | Presented with traumatic bereavement and associated behaviour dysregulation and peer socialisation challenges. Referral was made by the child's mother for emotional support and processing of multiple childhood adversities and life experiences, in addition to a family member's recent suicide. | Weekly 45–90-minute individual art therapy sessions over a 6-month period. Sessions were held both indoors and outdoors and focused on trauma and grief processing using naturally occurring materials and found waste, consistent with principles of ecopsychology. The service also offered referrals for food support, housing, benefits and accessing technology. | Qualitative: Clinical observations | Greater engagement in outdoor than indoor sessions. The child was more regulated processing bereavement in outdoor sessions. Child's mother shared that child had started discussing family member's death and engaged in memorial rituals together. His behavioural concerns decreased, and school attendance increased. | Participant initially met art therapist with silence and hostility, but became more engaged over time. |
| Weston (1988), USA | Inpatient locked child psychiatric unit. | 3 participants aged 5-8 years. 2 male, 1 female. | Various diagnoses including suicide attempts, depression, psychosis, conduct disorders, and learning disabilities. | Weekly art therapy sessions offered over 5 consecutive weeks. Young people were directed in creating a unique sock animal through foot tracing and storytelling, to promote integration of the self through control, increasing ego strength, and increasing bodily awareness consistent with psychodynamic approach. The psychiatric unit operated using behavioural management principles. | Qualitative: Clinical observations | Facilitated self-expression and sharing via turn-taking. | All children participated. Levels of engagement and materials used varied. Engagement improved over time, and children found the process comforting, reassuring and extremely rewarding. |
| Wolf (1975), USA | Non-profit secondary high school providing individualised services for handicapped adolescents. | 15-year-old male. | Presented with symptoms consistent with conduct disorder (e.g., antisocial behaviour, fire lighting, violent outbursts, absconding), and severe depression, in context of out-of-home care arrangement. No formal diagnosis reported as patient 'uncooperative' with diagnostic evaluation. | Twice weekly 45-minute individual art therapy sessions over a 6-month period. | Qualitative: Clinical observations | Participant showed improved emotional awareness, confidence and goal orientation, and a reduction in defence mechanisms of denial and distortion. | Participant developed a sense of pride and self-accomplishment, shown through increasing engagement and independently seeking time to make art. |
| Wyder (2019), France | Inpatient adolescent psychiatric unit. | 9 participants aged 15-17 years. 3 male, 6 female. | Various diagnoses including depression, drug addiction, bipolarity borderline disorders, autistic spectrum disorder, self-harm, obesity, and cognitive impairment. | Weekly 90-minute art therapy group workshops over a 3-month period. Sessions utilized a single specific theme, the house, to facilitate expression of traumatic experiences and self re-construction. Freely chosen modalities included painting and drawing, using acrylic colours, gouache, pencils, colours, graphite, coloured markers, rulers, erasers, graphite sticks, brushes, Chinese ink, ink stone, and two sizes of paper. | Mixed methods: Self-report measure of post-traumatic stress symptoms (Impact of Events Scale-Revised) and qualitative phenomenological coding of participant drawings, paintings, narratives, observations and 29-item semi-structured interviews. | Observational documentation of the body language and behaviour of the patients, as well as the interviews and fieldwork showed an improvement in expression of mental states, expression of painful past experiences, and potential improvements in mental health via positive self-evaluation. Too few participants completed the Impact of Events Scale-Revised to draw conclusions regarding post-traumatic stress symptoms. | N/R |

Abbreviations: ADHD=Attention Deficit Hyperactivity Disorder, DSM=Diagnostic and Statistical Manual of Mental Disorders, FASD=Foetal alcohol spectrum disorder, ICD= International Classification of Diseases, N/R= Not reported, PTSD= post-traumatic stress disorder.
